# Assessing availability, prices, and market share of quality-assured malaria ACT and RDT in the private retail sector in Nigeria and Uganda

**DOI:** 10.1186/s12936-024-04863-9

**Published:** 2024-02-06

**Authors:** Meley Woldeghebriel, Ezinne Aso, Erica Berlin, Chizoba Fashanu, Sylvia N. Kirumira, Felix Lam, Robert Mugerwa, Juliet Nakiganda, Tayo Olaleye, Jimmy Opigo, Funlola Osinupebi, Natalie Priestley, Rodger Stringham, Perpetua Uhomoibhi, Theodoor Visser, Abigail Ward, Owens Wiwa, Aaron Woolsey

**Affiliations:** 1 Clinton Health Access Initiative, Kampala, Uganda; 2 Clinton Health Access Initiative, Abuja, Nigeria; 3 Clinton Health Access Initiative, Boston, Massachusetts USA; 4 National Malaria Control Division, Ministry of Health, Kampala, Uganda; 5 National Malaria Elimination Programme, Abuja, Nigeria; 6 Virginia Commonwealth University, Richmond, Virginia USA

**Keywords:** Artemisinin-based combination therapy, Private sector, Drug shop, Clinic, Pharmacy

## Abstract

**Background:**

An estimated 50% of suspected malaria cases in sub-Saharan Africa first seek care in the private sector, especially in private medicine retail outlets. Quality of care in these outlets is generally unknown but considered poor with many patients not receiving a confirmatory diagnosis or the recommended first-line artemisinin-based combination therapy (ACT). In 2010, a subsidy pilot scheme, the Affordable Medicines Facility malaria, was introduced to crowd out the use of monotherapies in favour of WHO-pre-qualified artemisinin-based combinations (WHO-PQ-ACTs) in the private health sector. The scheme improved the availability, market share, and cost of WHO-PQ-ACTs in countries like Nigeria and Uganda, but in 2018, the subsidies were halted in Nigeria and significantly reduced in Uganda. This paper presents findings from six retail audit surveys conducted from 2014 to 2021 in Nigeria and Uganda to assess whether the impact of subsidies on the price, availability, and market share of artemisinin-based combinations has been sustained after the subsidies were reduced or discontinued.

**Methods:**

Six independent retail audits were conducted in private medicine retail outlets, including pharmacies, drug shops, and clinics in Nigeria (2016, 2018, 2021), and Uganda (2014, 2019, 2020) to assess the availability, price, and market share of anti-malarials, including WHO-PQ-ACTs and non-WHO-PQ-ACTs, and malaria rapid diagnostic tests (RDTs).

**Results:**

Between 2016 and 2021, there was a 57% decrease in WHO-PQ-ACT availability in Nigeria and a 9% decrease in Uganda. During the same period, non-WHO-PQ-ACT availability increased in Nigeria by 41% and by 34% in Uganda. The price of WHO-PQ-ACTs increased by 42% in Nigeria to $0.68 and increased in Uganda by 24% to $0.95. The price of non-WHO-PQ-ACTs decreased in Nigeria by 26% to $1.08 and decreased in Uganda by 64% to $1.23. There was a 76% decrease in the market share of WHO-PQ-ACTs in Nigeria and a 17% decrease in Uganda. Malaria RDT availability remained low throughout.

**Conclusion:**

With the reduction or termination of subsidies for WHO-PQ-ACTs in Uganda and Nigeria, retail prices have increased, and retail prices of non-WHO-PQ-ACTs decreased, likely contributing to a shift of higher availability and increased use of non-WHO-PQ-ACTs.

**Supplementary Information:**

The online version contains supplementary material available at 10.1186/s12936-024-04863-9.

## Background

Private sector health service delivery is an important component of many health systems in malaria-endemic countries and it is often the primary source of care for children under five with febrile illness [1, 2]. Each year, approximately 31% of over 200 million cases of malaria, and hundreds of millions of cases of non-malaria febrile illness seek care from the private sector, which includes private medicine retail outlets (PMRs) like drug shops and clinics [3]. Regulatory oversight of the private sector is often absent, inadequate, or not enforced in most malaria-endemic countries and little to no case data or drug consumption data are reported into public surveillance systems [1, 4]. Additionally, the quality of care provided to malaria patients is generally poor, with patients not receiving a confirmatory diagnosis or the appropriate treatment [1, 4–6].

Adoption of artemisinin-based combination therapy (ACT), the treatment recommended by the World Health Organization (WHO) for uncomplicated malaria in the private sector has been slow due to their high retail cost [6–10]. To improve the affordability and uptake of ACT in the private sector, low-cost artemisinin-based combinations were introduced in the private sector through an ex-factory subsidy scheme implemented in nine malaria-endemic countries: Cambodia, Ghana, Kenya, Madagascar, Niger, Nigeria, Tanzania, Uganda, Zanzibar [11, 12]. Coined the Affordable Medicines facility-malaria (AMFm), the Global Fund against TB, HIV and Malaria (GF) agreed to finance the majority of the cost for procurement of ACTs that met prequalification status by the WHO (WHO-PQ-ACTs). These WHO-PQ-ACTs were labelled with a green leaf logo and sold to approved importers who then leveraged existing private-sector distribution channels to deliver these artemisinin-based combinations to private sector retailers [13]. Due to the significant reduction in cost at import, the retail price of WHO-PQ-ACTs in the private sector was reduced by as much as a tenfold. As a result, private sector WHO-PQ-ACT availability increased from 9–27% at baseline to 53–83% at endline in six of the nine implementing countries [8, 13–17]. The private sector co-payment mechanism (CPM), which succeeded the AMFm and continued to use GF funds to make low-cost artemisinin-based combinations available to importers and distributors in the private sector, maintained or further improved WHO-PQ-ACT availability, market share, and pricing in Nigeria, Tanzania, and Uganda through 2015 [16]. In recent years, CPM subsidies for WHO-PQ-ACTs have dwindled. Nigeria and Kenya stopped the subsidy in 2018 altogether, while Uganda and Tanzania had reduced the subsidy, from 95 to 70% for all treatment pack sizes in Uganda and to 75% adult and 85% paediatric treatment pack sizes in Tanzania [16].

Contrary to the increase in uptake of ACT, following the AMFm and CPM, RDT uptake in the private sector did not improve after their introduction in the early 2000s [18]. Although many countries adopted the 2010 WHO policy that all suspected malaria cases receive a parasitological test to confirm a malaria diagnosis prior to treatment, testing for malaria primarily occurred in the public sector [3, 19, 20]. Consequently, many febrile patients seeking care in the private sector received ACT without a malaria diagnosis, often subsidized through the AMFm, leading to wastage of resources [15, 21–25]. Mathematical models suggested that an estimated 1.1 billion anti-malarials were taken by non-malaria febrile patients in 2016 [26]. Subsequently, several studies explored if and how RDTs could be introduced in the private sector [22, 24, 27, 28] often leading to mixed results with many patients receiving an anti-malarial despite a negative RDT result [7, 23, 29–32]. Large-scale efforts to improve the availability and use of RDTs in the private sector did not occur.

During AMFm, large-scale, nationally representative household and retail audits were conducted by ACTwatch/PSI to assess the AMFm’s impact on the availability and use of artemisinin-based combinations. Since the last ACTwatch nationwide household and retail surveys were completed in 2016, nationally representative retail audits have not been conducted to evaluate ACT and RDT availability, price, and market share in the private sector. This paper shows the results of six retail audits conducted in Uganda and Nigeria between 2016 and 2021 that aimed to track anti-malarial and RDT pricing, availability, and sales volumes among PMRs, and to determine if the impact of ACT subsidies on price, availability, and market share has been sustained.

## Methods

### Study design

Audits of child health commodities, including anti-malarials and RDTs, were conducted via six independent cross-sectional surveys in private medicine retail outlets (PMRs) in Nigeria (4 LGAs in Kano state and 4 LGAs in Lagos state in 2016, 2018, and 2021), and Uganda (79 districts in 2016, 58 districts in 2018, and 42 districts in 2020) to assess availability, market share, and pricing of malaria commodities in PMRs. Although the individual surveys were not designed as components in a longitudinal study, the objectives and methods of each of the six retail audit surveys were similar. When possible, the methodology followed that of the ACTwatch Outlet Surveys to enable comparisons over time with prior retail audit surveys [17]. Differences in the sampling strategies are explained below and in more detail in Additional file 1.

### Sampling strategy

In each audit, a multi-stage cluster sampling methodology was used based on sub-national units (e.g., Local Government Area (LGA) in Nigeria and district in Uganda). Study areas were identified as areas where data collection was logistically feasible, prior ACTwatch or other retail audit data had been collected and were considered to produce information to support a national understanding of ACT market dynamics in the private sector. Specific numbers and locations of LGAs or districts were selected for inclusion based on probability proportional to population size (PPS). In Nigeria and Uganda, national census enumeration areas (EAs) were stratified by urban vs. rural and selected EAs within LGAs and districts randomly, such that the ratio between urban and rural EAs reflected the approximate distribution of urban vs. rural households, based on the most recent census data. Based on information from national or local registries, previous surveys or lists, and using “snowball sampling”, PMRs were identified in each of the EAs. Snowball sampling was deployed because there were no available, up-to-date lists of PMRs due to frequent changes. The 2020 Uganda audit used a different sampling strategy and randomly sampled PMRs that were in the catchment areas of wholesalers that were part of the CPM distribution network. Differences between country-specific audits are explained in detail in Additional file 2.

### Sample size

A sample size of 400 PMRs was targeted in each retail audit in Uganda (2016 and 2018) and Nigeria (2016, 2018, and 2021), based on surveying logistics and available resources. The sample size of 400 PMRs was expected to produce an estimate of the percent of anti-malarial stocking PMRs with WHO-PQ-ACTs with 95% confidence interval of ± 5%. The 2020 Uganda audit did not have a target of 400 as PMRs were conveniently sampled based on a list of the distribution networks of importers across Uganda.

### Inclusion and exclusion criteria

PMRs including private for-profit, private not-for-profit, pharmacies, and drug shops that directly serve community members from the selected EAs were included in all six audits. Attendants in the PMRs were required to be 18 years or older to participate in the survey. PMRs had to meet at least one of the following inclusion criteria: (1) have one or more anti-malarials in stock at the time of the survey visit, (2) reportedly had one or more anti-malarials in stock in the previous three months, or (3) provide malaria blood testing (microscopy or RDT). PMRs that were visited but not open at the time of data collection were excluded. Informed consent was obtained from PMR providers who agreed to participate and met the inclusion criteria. PMR providers who did not provide informed consent were excluded from the study. Private outlets or facilities like tertiary hospitals, referral hospitals, and outlets not providing services to the public (e.g., army and military clinics) were excluded. No public facilities, community health workers, or grocery stores/kiosks were included.

### Data collection

For each of the six surveys, a team of trained data collectors from the areas in which the research took place used a SurveyCTO tool administered to the PMR provider, complemented by a visual inspection of store inventory. Data collectors asked shop attendants to bring their inventory of anti-malarials and RDTs to the front of the shop for visual inspection and data collection. Records of anti-malarial and RDT sales were also reviewed by data collectors if available. Photos of different RDT and ACT packs were taken to verify the commodity and identify variations of the green-leaf logo in circulation. The survey tool was administered in the English language; in instances where translation to a local language was required, the data collectors translated and explained the question to the provider. The survey collected data on antimalarial and RDT availability, stock levels, average re-stocking frequency, sales volumes (for the past day and over the past seven days), and retail price by brand, dose, and formulation. All six audits collected these data except the 2020 Uganda audit which did not collect data on sales volumes or prices of WHO-PQ-ACTs.

### Data analysis

Primary outcomes were descriptions of availability, price, and market share of WHO-PQ-ACTs, non-WHO-PQ-ACTs, other antimalarials, and RDTs by country and over time. All analyses were conducted using Stata 14.2 (Stata Corp), except for the 2020 Uganda audit, which was analyzed using Microsoft Excel. Analyses were stratified by state (Nigeria only), enumeration-level urban/rural designation, outlet type, and WHO-PQ-ACTs/non-WHO-PQ-ACTs. Brand, manufacturer, ingredient list, strength, pack size, and authentic green-leaf presence (verified and compared to the official ACT green-leaf logo), were extracted from the collected forms and images. Audited commodities were categorized into four age-weight bands based on ingredient strength and the number of tablets in the package to determine the total number of treatments: (i) infant/ < 5 kg; (ii) child/5-15 kg; (iii) junior/15-35 kg; (iv) adult/ > 35 kg. A WHO-PQ-ACT was defined as matching generic ingredients, strength, pack size, brand, and manufacturer of a WHO pre-qualified artemisinin-based combination as of each year the audits were conducted [33].

### Availability

Artemisinin-based combination availability was defined as the proportion of sampled outlets with at least one medicine (WHO-PQ-ACTs or non-WHO-PQ-ACTs) in stock on the day of the survey. Availability of WHO-PQ-ACTs and non-WHO-PQ-ACTs for each survey is presented descriptively. RDT availability trends were assessed descriptively.

### Price

Prices were adjusted to baseline year (2016 for Nigeria; 2014 for Uganda) US Dollars. First, prices were multiplied by the ratio of the national consumer price indices for 2018 and 2020 compared to the baseline year using International Monetary Fund International Fund Statistics [33]. Then, adjusted prices were multiplied by the annual average exchange rate for the baseline year [34]. Means are presented with a 95% confidence interval [35]. Prices were then summarized as volume-weighted means based on reported sales volume per treatment in the 7 days prior to the survey except for Uganda 2020 where data on volumes were not collected. Changes in mean pricing over time are generally non-linear and are presented descriptively.

### Market share of ACT and market share of anti-malarials

Market share of artemisinin-based combinations sold followed the methods of ACTwatch and was defined as the percentage of total ACT sales (sum of WHO-PQ-ACTs and non-WHO-PQ-ACTs treatment packs) that were WHO-PQ-ACTs (or non-WHO-PQ-ACTs). Market share of antimalarials sold was defined as the percentage of total anti-malarial sales over the past seven days (sum of WHO-PQ-ACTs, non-WHO-PQ-ACTs and other anti-malarials treatment packs) that were WHO-PQ-ACTs, non-WHO-PQ-ACTs or other anti-malarial treatment packs. Market share trends were assessed descriptively. The Nigeria 2018 and 2021 survey analysis applied sampling weights calculated as the inverse probability of an enumeration area being selected; all other surveys were designed to be self-weighting.

## Results

Across the six retail audits, 2,658 PMRs were surveyed. The types and locations of PMRs surveyed varied in each country with drug shops being the vast majority in each country (78.4%) (Table 1).

**Table 1:** PMRs sampled in Nigeria and Uganda

	Nigeria			Uganda		
Year	2016	2018	2021	2014	2018	2020
Shops surveyed	482	413	389	538	351	103
Urban	320	315	243	155	125	–
Rural	162	98	146	383	226	–
Drug shops	427	347	357	352	217	50
Clinics	0	0	0	146	123	21
Pharmacies	55	66	31	22	11	32
Not-for-profit clinic	0	0	0	18	0	0

### Availability of WHO-PQ-ACTs

**Fig. 1:**
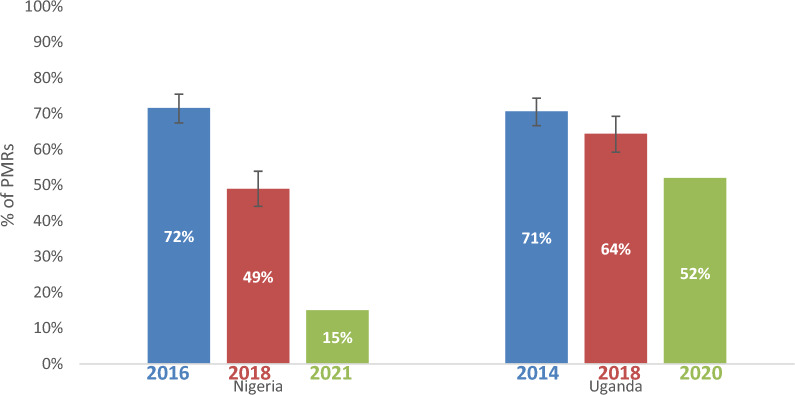
Availability of WHO-PQ-ACTs in Nigeria and Uganda 2014–2021

Nearly three-quarters of PMRs had WHO-PQ-ACTs in stock during the 2014/2016 baseline surveys in Nigeria and Uganda (Fig. 1). In Nigeria, WHO-PQ-ACTs were available in 72% (95% CI [67%–75%]) of sampled PMRs in 2016, 49% (95% CI [44%–54%]) in 2018, and in only 15% (95% CI [11%–19%]) of such PMRs in 2021. The difference in WHO-PQ-ACT availability between 2016 and 2018 was statistically significant (OR = 0.37, 95% CI [0.28–0.48]) as well as between 2016 and 2021 (OR = 0.07, 95% CI [0.05–0.09]). Across all three surveys in Nigeria, WHO-PQ-ACT availability was highest in pharmacies (84% in 2016; 74% in 2018; 20% in 2021) compared to drug shops (70% in 2016; 44% in 2018; 14% in 2021).

There was not a statistically significant decrease in WHO-PQ-ACT availability in pharmacies between 2016 and 2018, however, there was a statistically significant decrease between 2016 and 2021 (OR = 0.05, 95% CI [0.02–0.14]). In drug shops, there was a statistically significant decrease in WHO-PQ-ACT availability between 2016 and 2018 (OR = 0.33, 95% CI [0.24–0.44]), and between 2016 and 2021 (OR = 0.07, 95% CI [0.05–0.11]). In Uganda, WHO-PQ-ACTs were availablen in 71% of sampled PMRs (95% CI [67%–74%]) in 2014 and decreased to 64% of sampled PMRs (95% CI [59%–69%]) in 2018. In 2020, 52% of sampled PMRs in Uganda had WHO-PQ-ACTs in stock and availability was highest in pharmacies in 2014 (95%) and 2018 (73%), however in 2021 it was highest in clinics (76%) followed by drug shops (50%) and then pharmacies (41%). There was no statistically significant difference in WHO-PQ-ACT availability in Uganda between 2014 and 2018.

WHO-PQ-ACT availability also varied by geography and was higher in urban areas compared to rural areas in both countries in all surveys (Additional file 5).

### Availability of non-WHO-PQ-ACTs

**Fig. 2:**
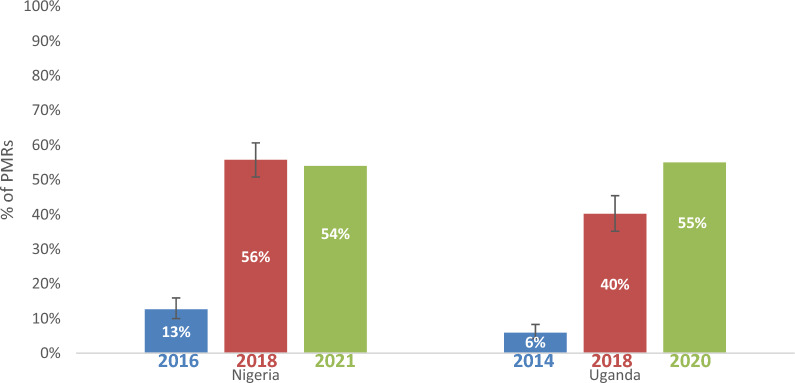
Availability of Non-WHO-PQ-ACTs in Nigeria and Uganda 2014–2021

Availability of non-WHO-PQ-ACTs varied over time in Nigeria and Uganda in sampled PMRs (Fig. 2). In Nigeria, non-WHO-PQ-ACT availability increased from 13% in 2016 (95% CI [10%–16%]) to 56% in 2018 (95% CI [51%–61%]) to 54% in 2021 (95% CI [48%–59%]). Availability was higher in pharmacies compared to drug shops in all survey years (40% vs 9% in 2014; 87% vs 50% in 2018; 74% vs 52% in 2021). Although not a primary indicator, in Nigeria imitation green leaf logos on non-WHO-PQ-ACTs were found in 18% of the PMRs surveyed in 2018 and in 11% of observed ACTs in 2021. In Uganda, the availability of non-WHO-PQ-ACTs in sampled PMRs increased from 6% (95% CI [4%–8%]) in 2014 to 40% (95% CI [35%–45%]) in 2018. In 2020, 55% of PMRs in Uganda had non-WHO-PQ-ACTs in stock. Availability was highest in pharmacies in 2014 (50%) and 2018 (55%), however in 2021 availability was highest in private clinics (81%). Non-WHO-PQ-ACT availability remained higher in urban areas compared to rural areas in all audits in Uganda and Nigeria (Additional file 6).

### Price of WHO-PQ-ACTs

**Fig. 3:**
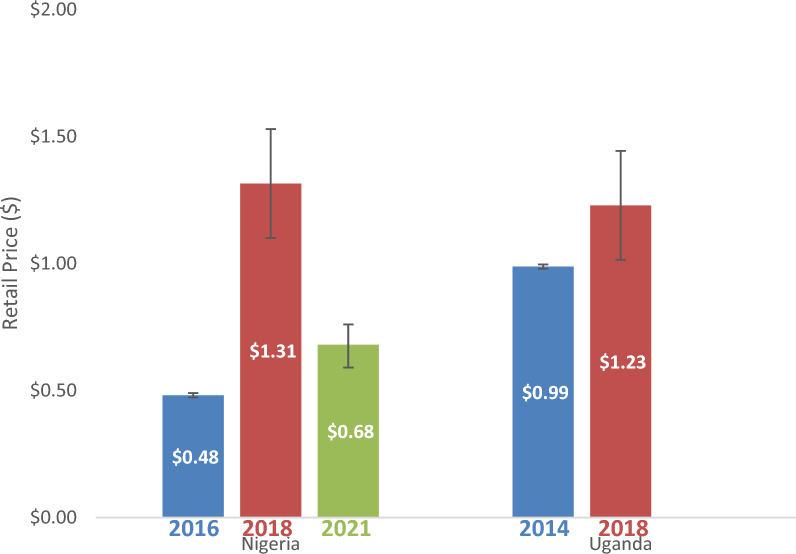
Average Retail Price of WHO-PQ-ACTs in Nigeria and Uganda 2014–2021

In Nigeria, retail prices were collected for 6,036 WHO-PQ-ACT treatment sales in the seven days prior to the survey in 2016. Retail prices were collected for 3,061 treatment sales in 2018 and 1,737 treatment sales in 2021. In Uganda, retail prices were collected for 7,195 treatment sales in the last seven days in 2014 and 2,201 treatment sales in 2018. In Nigeria, volume-weighted average price of WHO-PQ-ACTs increased from $0.48 (95% CI [$0.47–$0.49]) in 2016 to $1.31 (95% CI [$1.10–$1.53]) in 2018 and then declined to $0.68 (95% CI [$0.60–$0.77]) in 2021 (Fig. 3). Although overall volume-weighted average price of WHO-PQ-ACTs declined in 2021, this was only observed in Kano state (from $0.72 in 2018 to $0.63 in 2021); in Lagos state, the volume-weighted average price of WHO-PQ-ACTs increased from $1.59 in 2018 to $2.37 in 2021. (In Uganda, the volume-weighted average price of WHO-PQ-ACTs in Uganda increased from $0.99 (95% CI [$0.97–$1.00]) in 2014 to $1.23 (95% CI [$1.20–$1.26]) in 2018. The retail price of WHO-PQ-ACTs also varied by urban/rural setting and was higher in urban areas compared to rural areas in both countries and across all periods (Additional file 7).

### Price of non-WHO-PQ-ACTs

**Fig. 4:**
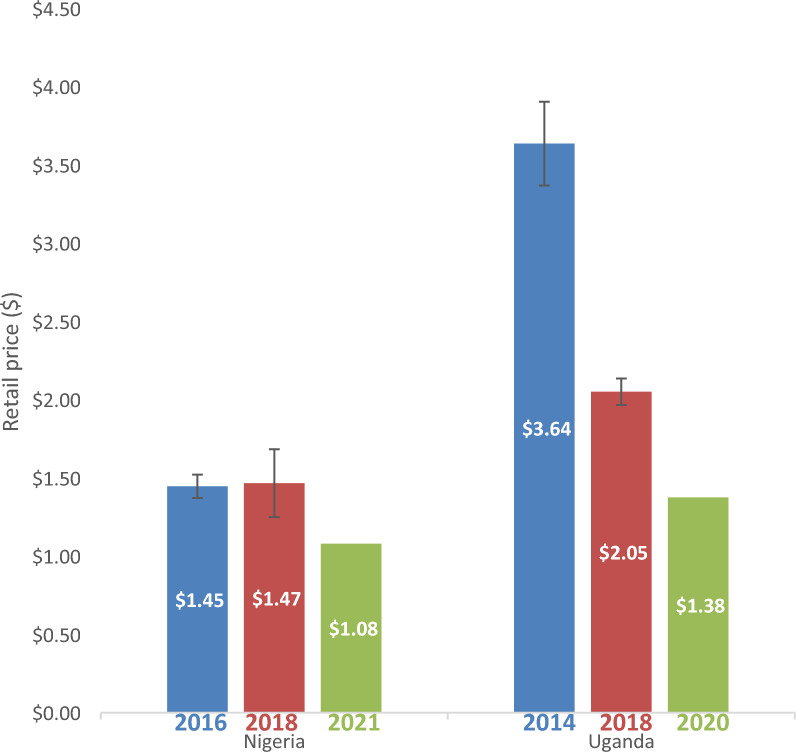
Average Retail Price of non-WHO-PQ-ACTs in Nigeria and Uganda 2014–2021

In Nigeria, in 2016, retail prices were collected for 528 treatment sales in the seven days prior to the survey, 2,938 treatment sales in 2018, and 9,197 treatment sales in 2021. In Uganda, retail prices for non-WHO-PQ-ACT were collected for 243 treatment sales in 2014 and 536 treatment sales in 2018. In Nigeria, volume-weighted average prices of non-WHO-PQ-ACTs slightly increased from $1.45 (95% CI [$1.37–$1.52]) in 2016 to $1.47 (95% CI [$1.25–$1.68]) in 2018 and then declined to $1.08 (95% CI [$0.88–$1.29]) in 2021 (Additional file 8). Similarly in Uganda, the volume-weighted average price of non-WHO-PQ-ACTs fell from $3.64 in 2014 (95% CI [$3.37–$3.91]) to $2.05 (95% CI [$1.97–$2.14]) in 2018 to an unweighted average price of $1.38 in 2020. The retail price of non-WHO-PQ-ACTs also varied by urban/rural setting and was higher in urban areas compared to rural areas in both countries across all periods (Fig. 4).

### Market share of WHO-PQ-ACTs among all ACTs and among all anti-malarials

**Fig. 5:**
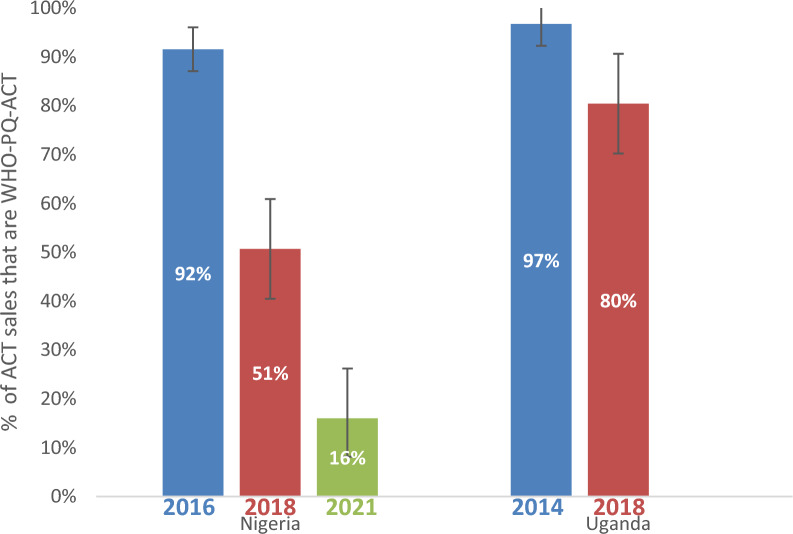
Market share of WHO-PQ-ACTs among ACTs sold in Nigeria and Uganda 2014–2021

Over 90% of ACT sales in the seven days prior to the survey were WHO-PQ-ACTs during the 2014/2016 baseline surveys in Nigeria and Uganda. In subsequent years, this proportion declined in both Uganda and Nigeria (Fig. 5). In Nigeria this proportion decreased from 92% in 2016 (95% CI [87%–96%]) to 51% in 2018 (95% CI [40%–61%]) to 16% in 2021 (95% CI [4%–28%]. In Uganda, the proportion decreased from 97% in 2014 (95% CI [95%–99%]) to 80% in 2018 (95% CI [73%–88%]) (Additional file 9).

**Fig. 6:**
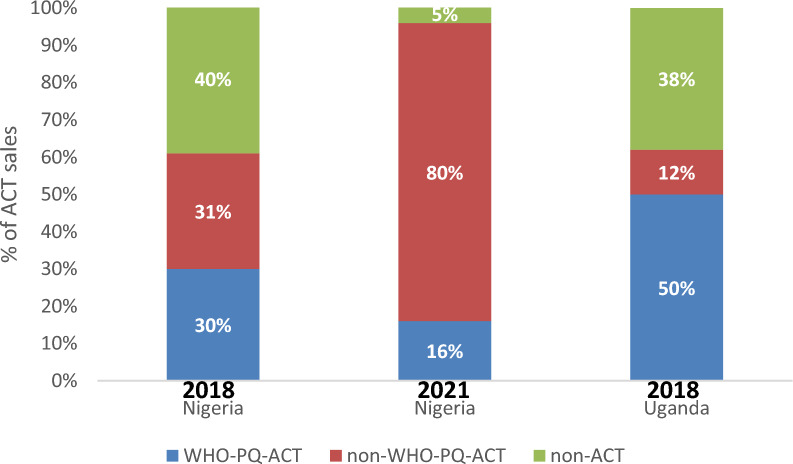
Market share of WHO-PQ-ACTs among all antimalarials sold in Nigeria and Uganda 2018–2021

The market share of WHO-PQ-ACTs, non-WHO-PQ-ACTs, and non-artemisinin-based anti-malarials among all anti-malarials sold was also assessed. In Nigeria, in 2018, non-artemisinin-based anti-malarials had the highest market share (40%, 95% CI [25%–54%]) compared to non-WHO-PQ-ACTs (31%, 95% CI [23%–38%]) and WHO-PQ-ACTs (30%, 95% CI [19%–41%]). In 2021, market share shifted to mostly non-WHO-PQ-ACTs (80%, 95% CI [69%–91%]), followed by WHO-PQ-ACT market share (16%, 95% CI [4%, 27%]) and non-ACT market share to 5% (95% CI [0%–11%]). In Uganda, WHO-PQ-ACT market share was highest in 2014 (50%, 95% CI [37%–63%]), followed by non-artemisinin-based market share (38%, 95% CI [25%–50%]) and then non-WHO-PQ-ACT (12%, 95% CI [8%–17%]) [Fig. 6].

### Availability of RDTs

**Fig. 7:**
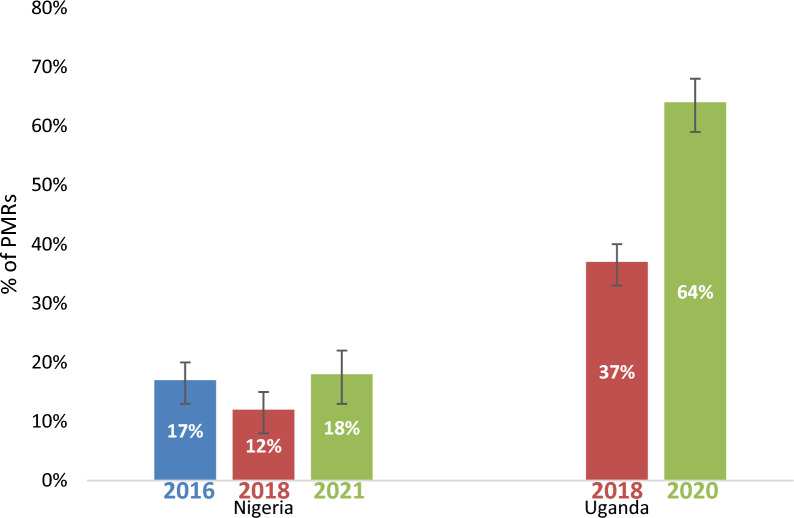
RDT Availability in Nigeria and Uganda 2016–2021

RDT availability either increased or remained the same over time in Nigeria and Uganda in sampled PMRs. In Nigeria, RDTs were available in 17% of sampled PMRs (95% CI [14%–21%]) in 2016, 12% of sampled PMRs in 2018 (95% CI [9%–16%]) and 18% of sampled PMRs (95% CI [14%–23%]) in 2021 (Additional file 10). In Uganda, RDT were available in 37% of sampled PMRs (95% CI [32%–42%]) in 2018 and increased to 56% in 2020 (Fig. 7). RDT availability also varied by urban/rural settings. In Nigeria, RDT availability was higher in rural areas compared to urban areas across all periods, unlike Uganda where RDT availability was higher in urban areas compared to rural areas in 2018. No RDT data was collected in Uganda in 2014 and data were not stratified by geography in 2020.

### Prices of RDTs

**Fig. 8:**
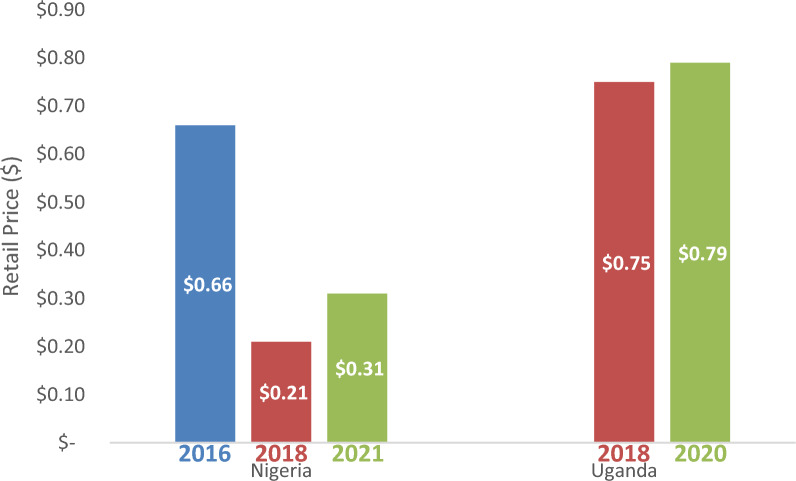
RDT Prices in Nigeria and Uganda 2016–2021

In Nigeria, the volume-weighted mean price of RDTs decreased from $0.66 in 2016 (95% CI [$0.66–$0.66]) to $0.21 in 2018 (95% CI [$0.13–$0.30]), but then increased to $0.31 in 2021 (95% CI [$0.28–$0.34]). In Uganda, the volume-weighted mean price of RDT remained the same: $0.75 in 2018 (95% CI [$0.72–$0.77]) and $0.79 in 2020 (Fig. 8) (Additional file 11). RDT price also varied by geography and was higher in urban areas compared to rural areas across all surveys where data were collected.

## Discussion

This paper presents data on WHO-PQ-ACT and non-WHO-PQ ACT availability, market share and price, and RDT price and availability in the PMRs from six retail audit surveys in two countries (Nigeria and Uganda), conducted between 2014 and 2021. These retail audit surveys covered a period when subsidies that induced WHO-PQ-ACT uptake in the private retail sector were being reduced or discontinued in Uganda and Nigeria as of 2018. The findings show that across all surveys in both countries, the average retail prices of WHO-PQ-ACTs increased, and availability and market share decreased over time (apart from the 2021 survey in Uganda where data on WHO-PQ-ACT retail price and market share were not collected). In Nigeria, WHO-PQ-ACT prices decreased in 2021 compared to 2018. However, this decline in price was only found among PMRs in Kano state, while in Lagos state, the average retail price increased from 2018 to 2021. This may be a result of the relatively high price of WHO-PQ-ACTs in Kano state in 2018, since the CPM subsidy scheme just ended and that the 2018 survey was conducted right after the end of the CPM. The decline may also be partially explained due to leakages of donor funded artemisinin-based combinations meant to be distributed for free in the public sector to the private sector.

During the 2014–2021 period, the availability and market share of non-WHO-PQACTs increased in both countries. The termination of the CPM subsidies on WHO-PQ-ACTs in Nigeria and the subsidy reduction in Uganda likely contributed to the observed price increases in WHO-PQ-ACTs and their subsequent decline in market share. In Uganda, where the subsidy is still in place, there was a smaller reduction in WHO-PQ-ACT availability and market share over time. Despite the shift from WHO-PQ-ACTs to non-WHO-PQ-ACTs, this did not negatively affect the overall market share of all ACTs among all anti-malarials sold; in Nigeria, overall ACT market share in 2018 was 61% and in 2021, it was 96%. Thus, the termination of ACT subsidies in Nigeria is not causing a shift towards the use of monotherapies.

Compared to the last nationally representative retail audits conducted in Uganda and Nigeria by ACTwatch in 2015, this study found lower and declining availability and market share of WHO-PQ-ACTs. In 2015, ACTwatch found high availability of WHO-PQ-ACTs (over 84% in Nigeria and 77% in Uganda), market share (35% in Nigeria, 48% in Uganda), and decreasing price (in Nigeria from $1.34 in 2011 to $1.24 in 2015 and in Uganda from $1.86 in 2011 to $1.48 in 2015) in the private sector [15]. With respect to non-WHO-PQ-ACTs, the data show high and increasing availability and market share of non-WHO-PQ-ACTs. In 2015, ACTwatch non-WHO-PQ availability was 48% in Nigeria and 38% in Uganda, market share was 11% in Nigeria and 17% in Uganda, and price increased in Nigeria from $3.21 in 2011 to $2.24 in 2015 and in Uganda from $3.91 in 2011 to $2.96 in 2015. During the peak of the ACT subsidy period, retail prices of WHO-PQ-ACTs in the private sector was and remained low, with high availability and market share. Once subsidies began to diminish, there was a decline in WHO-PQ-ACT market share, and an increase in retail prices as the data in this paper show. Results presented in this paper are similar to the results of retail audits conducted in 2018 in six states in Nigeria that found lower availability of WHO-PQ-ACTs compared to non-WHO-PQ-ACTs. Unlike our results, the 2018 study found no discernable differences between the retail prices of WHO-PQ-ACTs and non-WHO-PQ-ACTs, although non-WHO-PQ-ACT prices were obtained for one brand, Lonart [26].

The increase in availability of non-WHO-PQ-ACTs following the reductions or termination of subsidies for WHO-PQ-ACTs, may raise concerns around the quality of artemisinin-based combinations that are not evaluated by the WHO prequalification programme [35]. To evaluate the quality of non-WHO-PQ-ACTs, samples of the most commonly found non-WHO-PQ-ACTs brands in surveyed PMRs during the 2018 and 2021 retail audits in Nigeria and the 2018 retail audit in Uganda were purchased and submitted for laboratory testing. Ingredients in a pill from each sample were tested against reference samples to measure the concentration of active pharmaceutical ingredients using a common method, called reverse-phase high-performance liquid chromatography (HPLC). Since the cause of any potential adverse findings (i.e., transport or storage challenges could have contributed to any degradation) could not be determined, the results of the testing are not presented in this paper. However, testing showed that most of the commonly found brands of the non-WHO-PQ-ACT samples contained the expected concentration of active pharmaceutical ingredients in the treatment of uncomplicated malaria which suggests that the most popular brands sold by PMRs appear to meet industry standards [26]. Full reports available in Additional files 3 and 4. Further inquiries with the national regulatory authorities also found that most of the non-WHO-PQ-ACTs were approved to be imported and sold in Uganda and Nigeria. Thus, the shift to lower priced non-WHO-PQ-ACTs may not necessarily mean that these artemisinin-based combinations are of poor quality. Yet, given the variety of brands and types of ACT available in the private sector, post-market surveillance efforts should focus on evaluating the quality of ACTs in the private sector.

There were mixed results around the availability of RDTs. Availability in Nigeria remained low, most likely due to low demand, with 12–18% of sampled PMRs stocking RDTs between 2016 and 2021. In contrast, in Uganda, RDT availability increased from 37% in 2018 to 64% in 2020. The higher RDT availability in Uganda may be attributed to government policies favoring RDT use, ranging from pre-negotiated agreements with importers to lower prices for RDT importers, waivers of value-add tax (VAT) for importers of quality-assured RDTs, to interventions sensitizing communities, training providers on the test and treat policy, and increasing distribution networks for malaria RDTs through peer detailing. Results in Nigeria were consistent with other audits showing low availability of RDTs [17, 26]. Operational research studies have shown that low availability of RDTs in the private retail sector in Nigeria and other countries may likely be attributed to a lack of trust in the RDT result by both patients and providers, and a preference of relying on presumptive diagnosis of malaria instead [23].

### Limitations

There are limitations in how the retail audits were designed and implemented. Unlike the ACTwatch surveys, which were broader in scope and included more providers, these audits included only specific regions of each country and thus were not nationally representative. Due to a lack of registries or lists of PMRs, a snowball sampling approach was selected and could have introduced a potential sampling bias, and therefore generalizability of results. In addition, although we physically inspected the commodities, the audits relied on self-reported data from outlet owners and attendants which may introduce positive response bias particularly on retail prices and sales of commodities. Moreover, while data collectors collected photos of artemisinin-based combination brands and packs to verify the data (e.g., to confirm the green-leaf logos are official and not counterfeit), this was not always consistently done which may have influenced the data accuracy. Additionally, although the objectives of the six audits were similar there were differences in the methodologies which limited direct comparison for certain research questions. For example, the 2020 Uganda audit used a different sampling approach, resulting in a small sample size, no distinction between rural and urban PMRs, no data on sales volumes, and limited data collection to subsidized WHO-PQ-ACTs (not all ACTs) due to the objectives of the CPM compliance audit. The 2020 audit in Uganda was conducted in districts where the CPM distributors were active, suggesting a higher likelihood of PMR stocking WHO-PQ-ACTs compared to other non-CPM districts.

## Conclusion

Investments from subsidy schemes like AMFm/CPM have improved the availability and price of WHO-PQ-ACTs and raised the profile, availability, and access to artemisinin-based combinations in private sector settings in Nigeria and Uganda. With the reduction or termination of these subsidies, WHO-PQ-ACTs have seen their market advantage and gains diminish. Meanwhile, non-WHO-PQ-ACTs have become more readily available and less expensive to retail customers. National regulatory authorities should prioritize routine monitoring of artemisinin-based combinations sold in the private health sector to ensure patients are provided appropriate and effective medicines. Further research to understand why providers in certain geographies like Uganda are more likely to stock and conduct malaria RDTs can help inform if and how malaria testing can improve malaria case management in the private sector.

### Supplementary Information

**Additional file 1.** Sampling strategy by country and year.

**Additional file 2.** Scopes of retail audits by country and year.

**Additional file 3. **ACT Testing Results 2018.

**Additional file 4. **ACT Testing Results 2021.

**Additional file 5. **WHO-PQ-ACT availability by country and year.

**Additional file 6. **Non-WHO-PQ-ACT availability by country and year.

**Additional file 7. **Mean price of WHO-PQ-ACTs by country and year.

**Additional file 8. **Mean price of Non-WHO-PQ-ACTs by country and year.

**Additional file 9: Table S6.** Market share of WHO-PQ-ACTs by country and year.

**Additional file 10: Table S7. **RDT Availability by country and year.

**Additional file 11: Table S8.** Mean price of RDTs by country and year.

## Data Availability

The data set analysed for this study is available from the corresponding author (Meley Woldeghebriel), upon request.
