# Supplementary material for: Assessing availability, prices, and market share of quality-assured malaria ACT and RDT in the private retail sector in Nigeria and Uganda
Source: Malar J. 2024 Feb 6;23:41. doi: 10.1186/s12936-024-04863-9 (PMC10848491; doi:10.1186/s12936-024-04863-9)
Supplement: Supplementary file 1 — Additional file 1. Sampling strategy by country and year. [file 12936_2024_4863_MOESM1_ESM.docx]

### Additional File 1: Sampling strategy by country and year

### Nigeria

In 2016, 2018 and 2021, the audits were conducted in two states (Kano and Lagos). Sample LGAs were selected based on the number of census EAs such that they were representative of the urban/rural distribution. Approximately four outlets per rural EA and five to eight outlets per urban EA were sampled using a snowball sampling approach. Samples of the most commonly found non-WHO-PQ-ACTs in surveyed PMRs were collected during the 2018 and 2021 audits.

### Uganda

In 2018, the audit was conducted in 58 districts. Based on EAs from the 2002 Uganda census, 168 EAs were required for a representative sample and were stratified into regions as defined by Uganda’s 2011 DHS (Kampala, Central 1, Central 2, East Central, Eastern, Karamoja, Northern, West Nile, Western, and Southwest). PMRs were identified via snowball sampling. In 2020, the audit was conducted in 54 districts. The NMCD conducted a compliance audit and surveyed first-line buyers who were part of the Co-Payment Mechanism and second-line buyers (wholesalers that were listed by the FLBs as in their distribution networks). In 2020, PMRs were included in the audit and randomly sampled based on the SLBs who indicated the outlets were within their catchment areas and snowball sampling.
