# Supplementary material for: Assessing availability, prices, and market share of quality-assured malaria ACT and RDT in the private retail sector in Nigeria and Uganda
Source: Malar J. 2024 Feb 6;23:41. doi: 10.1186/s12936-024-04863-9 (PMC10848491; doi:10.1186/s12936-024-04863-9)
Supplement: Supplementary file 2 — Additional file 2. Scopes of retail audits by country and year. [file 12936_2024_4863_MOESM2_ESM.docx]

### Additional File 2: Scopes of retail audits by country and year

The table below summarizes the scopes of each audit by country, locality, timeframe, type of PMR, sampling strategy, and type of data collected.

| **Country** | **Area Covered** | **Data collection period** | **Type of PMR** | **Sampling Strategy** | **Sample** | **Data collected** |
| --- | --- | --- | --- | --- | --- | --- |
| Uganda | 89 districts | October 16-December 5, 2014 | Drug Shop;  Pharmacy;  Private clinic/doctor; not-for-profit clinics | Multi-stage cluster sampling. PMRs in select EAs were identified using a snowball approach. | 538 | Price, pack size, market share, and availability of antimalarial commodities (by brand, dose, and formulation) |
| Uganda | 58 districts | November 13 – December 7, 2018 | Drug Shop;  Pharmacy;  Private clinic/doctor | Multi-stage cluster sampling. PMRs in select EAs were identified using a snowball approach. | 351 | Price, pack size, market share, and availability of antimalarial and RDT commodities (by brand, dose, and formulation) |
| Uganda | 42 districts | November 16-30, 2020 | Drug shop; clinic; pharmacy | Convenient sampling of PMRs within the distribution networks of first line and second line buyers in the CPM | 103 | Price, pack size and brands of co-paid ACTs, other antimalarials, and RDTs |
| Nigeria | 2 states | April 6-29, 2016 | Drugshop;  Pharmacy | Multi-stage cluster sampling methodology. PMRs in select EAs were identified using a snowball approach. | 482 | Price, pack size, market share, and availability of antimalarial and RDT commodities (by brand, dose, and formulation) |
| Nigeria | 2 states | November 23-December 6, 2018 | Drugshop;  Pharmacy | Multi-stage cluster sampling methodology. PMRs in select EAs were identified using a snowball approach. | 413 | Price, pack size, market share, and availability of antimalarial and RDT commodities (by brand, dose, and formulation) |
| Nigeria | 2 states | September 27 -October 18, 2021 | Drugshop;  Pharmacy | Multi-stage cluster sampling methodology. Retail shops in select EAs were identified using a snowball approach. | 389 | Price, pack size, market share, and availability of antimalarial and RDT commodities (by brand, dose, and formulation) |
