# Supplementary material for: Assessing availability, prices, and market share of quality-assured malaria ACT and RDT in the private retail sector in Nigeria and Uganda
Source: Malar J. 2024 Feb 6;23:41. doi: 10.1186/s12936-024-04863-9 (PMC10848491; doi:10.1186/s12936-024-04863-9)
Supplement: Supplementary file 3 — Additional file 3. ACT Testing Results 2018. [file 12936_2024_4863_MOESM3_ESM.pdf]

Analysis of ACT Field Samples

August 1, 2019

Rodger W. Stringham

Workers for the Clinton Health Access Initiative (CHAI) collected various samples of Artemisinin Combination Therapy (ACT) packages from marketplaces in Uganda and Nigeria. These samples were submitted to Medicines for All Institute (M4All) for analysis. CHAI was concerned that since the market had moved away from requiring SRA-qualified suppliers that many of the products might be fake or sub-standard. Samples were collected not in a representative way to draw conclusions regarding market integrity, but as a first pass to see if a problem might exist, justifying further investigation. As such the analysis of the samples did not need to be performed at a WHO PQ qualified laboratory. These samples were then submitted to M4All for preliminary evaluation. Reference samples of the APIs were kindly provided by Mangalam Drugs Company. In the tables below Claim contents are in mg per pill.

Table 1. Samples of DHA-Piperaquine phosphate ACT Formulations

Sample #	Manufacturer	Brand	Lot #	Claim DHA	Claim PipQ Phos
DP1	Bliss	P-Alaxin	H1AFN002	40	320
DP2	Bliss	P-Alaxin	H1AFN008	40	320
DP3	Bliss	P-Alaxin	H1AFN076	40	320
DP4	Bliss	P-Alaxin	H1AFN079	40	320
DP5	Bliss	P-Alaxin	H1AFN081	40	320

Table 2. Samples of Artemisinin-Piperaquine ACT Formulations

Sample #	Manufacturer	Brand	Lot #	Claim Arte	Claim PipQ
AP1	Artepharm	Artequick	20180702	62.5	375
AP2	Artepharm	Artequick	20180702	62.5	375
AP3	Artepharm	Artequick	20170902	62.5	375

Table 3. Samples of Artesunate-Amodiaquine ACT Formulations

Sample #	Manufacturer	Brand	Lot #	Claim AQ	Claim AS
AA1	Front	Camosunate	110917	300	100
AA2	Front	Camosunate	120917	300	100
AA3	Front	Camosunate Pediatric	140917	75	25
AA4	Front	Camosunate Children	391117	150	50
AA5	Front	Camosunate Junior	1707818	300	100
AA6	Sanofi	ASAQ Winthrop	6MA455	270	100

Table 4. Samples of Artemether-Lumefantrine ACT Formulations

Sample #	Manufacturer	Brand	Lot #	Claim ARM	Claim Lum
1	Novartis	Coartem	KF167	80	480
2	Novartis	Coartem	KF555	80	480
3	Bliss	Lonart DS	H1AFM129	80	480
4	Bliss	Lonart DS	H1AFM125	80	480
5	Bliss	Lonart Tablets	LRC054	20	120
6	Bliss	Lonart Tablets	LRC040	20	120
7	Bliss	Lonart Tablets	H1AFJ122	20	120
8	Bliss	Lonart Tablets	H1AFJ154	20	120
9	Bliss	Lonart Tablets	H1AFJ151	20	120
10	Teka *(for Afirst)	Bleortem	T18234	20	120
11	Laborate	Havax	18HX14	20	120
12	Laborate	Havax	18HX09	20	120
13	Laborate	Havax Forte	18HX04	80	480
14	Jiangsu Ruinian Qi	Clartem-DS	180521	80	480
15	Jiangsu Ruinian Qi	Clartem-DS	171123	80	480
16	Shalina	Dispersible	J8005	20	120
17	Shalina	Shal'Artem Forte	J8025	20	120
18	Shalina	Shal'Artem	J8007	80	480
19	Cipla	Lumartem	QK90094	20	120
20	Agog	Co-Mether	T86012	20	120
21	Agog	Co-Mether	T85061	20	120
22	Astra Lifecare	Lumether	53	20	120
23	Elbe	Amatem Forte Softgel	M18E001	80	480
24	Elbe	Amatem Forte Softgel	M18E002	80	480
25	Elbe	Amatem Softgel	S17K158	20	120

The samples highlighted in yellow were identified as ‘dissolvable’ gel caps.

Prior experience (and initial method development) indicated that a single method would not address all formulation samples. The intent then became the development of four methods that could assay both components of each ACT simultaneously. This was expected to be complicated by the relative content of the APIs strongly favoring the base component, further exacerbated by the relatively low UV absorbance of the artemisinin-based APIs.

A review of the literature describing ACT analyses indicated that many authors ended up using C-18 columns with acetonitrile vs. pH 3 phosphate buffers. Previous work at CHAI laboratories indicated that the bases in the ACTs are strongly affected by mobile phase and sample diluent pH changes. It had also been observed that the HPLC peaks

of the bases may overwhelm the peaks of the artemisinin derivatives such that resolution of these components may need to be better than otherwise expected. Preliminary tests of the various samples indicated that different assay conditions would be needed for each different ACT formulation.

Artemether-Lumefantrine Samples

HPLC Conditions

Column:	Agilent Extend-C18; 250 X 4.6 mm; 5 *u*M particles
Mobile phase:	(80:20) Acetonitrile:0.1% H_3PO_4 in water
Flow rate:	1.5 mL/min
Temperature:	30°C
Detection:	UV at 210 nm
Retention times:	Lumefantrine = 2.4 minutes; artemether = 5.1 minutes

Initial sample preparation targeted a single solution with lumefantrine at 5 mg/mL and artemether at 1 mg/mL in mobile phase. Lumefantrine did not dissolve completely at this concentration. Reducing the concentration to 1 mg/mL allowed complete dissolution of lumefantrine but the resultant artemether peak areas were too small to allow accurate quantitation. This forced the conclusion that separate runs and sample preps would be needed to analyze both APIs in the pill.

Separate sample preparations were then made, with a target of 1 mg/mL for lumefantrine and 2 mg/mL for artemether. At the target concentration of the artemether, much of the lumefantrine base did not dissolve and the extract was filtered before analysis. This approach assumes that undissolved lumefantrine would not interfere with artemether dissolution and analysis.

A single pill from each sample was weighed and then manually crushed. Based on the pill weight and the claimed API content a weight% of API in the crushed material was projected and used to calculate the amount of crushed material to be extracted for API analysis. With this approach the content determined is based on the entire pill weight. Samples were sonicated for 20 minutes with 10 mL mobile phase and filtered prior to injection. Samples with a claim of 20 mg artemether were prepared by extracting an entire pill. An injection volume of 1 *u*L was used for lumefantrine assay while 10 *u*L was used for artemether.

The ‘dissolvable’ gel caps proved problematic as the gel portion did not completely dissolve in mobile phase, neat ACN, water or any other solvent tested. As such, a representative sample of these formulations would not be possible.

Chromatogram of Sample for Lumefantrine Analysis

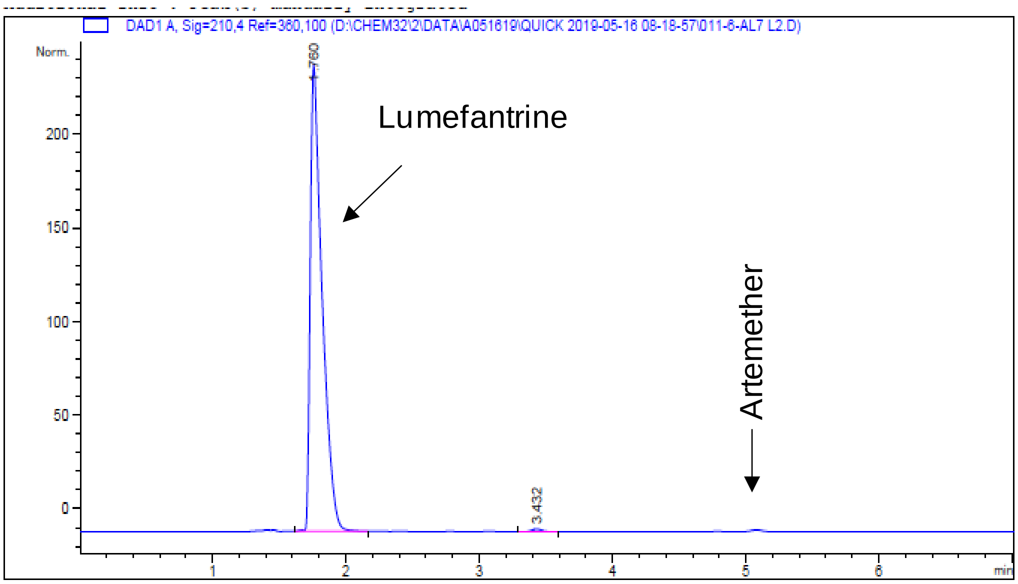

Note that the peak at 3.4 minutes is extracted from the plastic vials used for sample prep. The barely detectable peak at 5.0 corresponds to artemether.

Chromatogram of Sample for Artemether Analysis

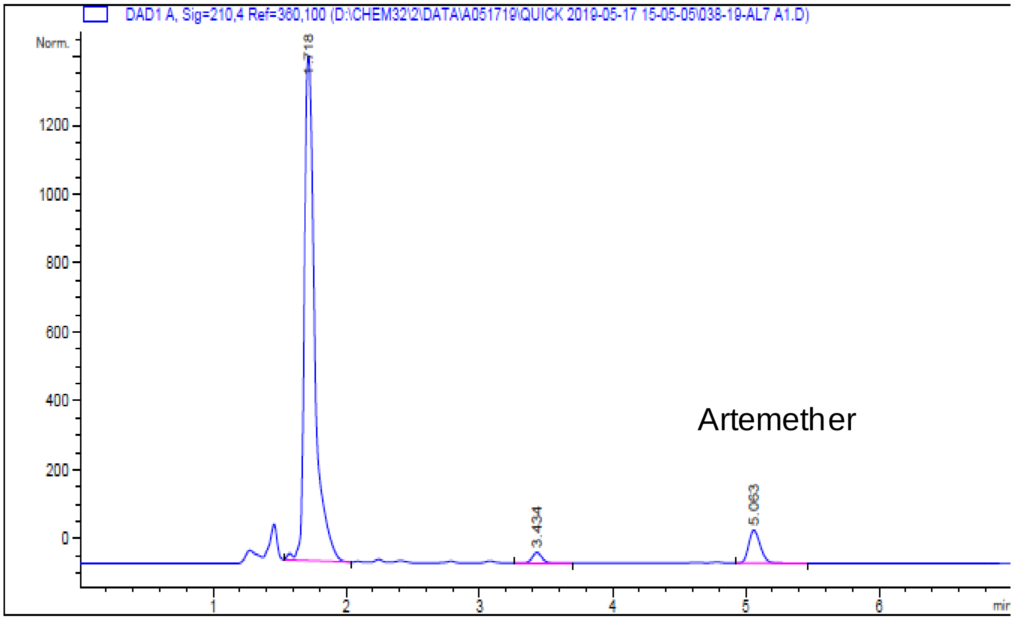

This approach worked quite well. Almost all samples met their claimed content for both lumefantrine and artemether depending on the acceptability range. If 90% of label is considered passing only one sample failed both APIs and two others contained 87% of the label lumefantrine.

Table 5. Artemether-Lumefantrine Results

Manufacturer	Brand	Lot #	Claim ARM	Claim Lum	Found Arm	Found Lum	%ARM	% Lum
Novartis	Coartem	KF167	80	480	78.9	478	98.6%	99.6%
Novartis	Coartem	KF555	80	480	78.0	453	97.5%	94.4%
Bliss	Lonart DS	H1AFM129	80	480	79.6	478	99.5%	99.6%
Bliss	Lonart DS	H1AFM125	80	480	80.9	475	101.1%	99.0%
Bliss	Lonart Tablets	LRC054	20	120	19.9	118	99.5%	98.3%
Bliss	Lonart Tablets	LRC040	20	120	19.5	114	97.5%	95.0%
Bliss	Lonart Tablets	H1AFJ122	20	120	19.6	125	98.0%	104.2%
Bliss	Lonart Tablets	H1AFJ154	20	120	19.9	122	99.5%	101.7%
Bliss	Lonart Tablets	H1AFJ151	20	120	20.7	118	103.5%	98.3%
Teka *(for Afirst)	Bleortem	T18234	20	120	17.1	101	85.5%	84.2%
Laborate	Havax	18HX14	20	120	20.6	117	103.0%	97.5%
Laborate	Havax	18HX09	20	120	20.2	104	101.0%	86.7%
Laborate	Havax Forte	18HX04	80	480	80.6	437	100.8%	91.0%
Jiangsu Ruinian Qi	Clartem-DS	180521	80	480	80.7	416	100.9%	86.7%
Jiangsu Ruinian Qi	Clartem-DS	171123	80	480	81.8	478	102.3%	99.6%
Shalina	Shal'Artem Dispersible	J8005	20	120	19.4	121	97.0%	100.8%
Shalina	Shal'Artem Forte	J8025	20	120	20.1	124	100.5%	103.3%
Shalina	Shal'Artem	J8007	80	480	81.9	477	102.4%	99.4%
Cipla	Lumartem	QK90094	20	120	20.1	119	100.5%	99.2%
Agog	Co-Mether	T86012	20	120	20.2	122	101.0%	101.7%
Agog	Co-Mether	T85061	20	120	20.4	118	102.0%	98.3%
Astra Lifecare	Lumether	53	20	120	19.2	110	96.0%	91.7%

Artesunate-Amodiaquine Samples

HPLC Conditions

Column:	Agilent Extend-C18; 250 X 4.6 mm; 5 *u*M particles
Mobile phase:	(60:40) Acetonitrile:0.1% H_3PO_4 in water
Flow rate:	1.5 mL/min
Temperature:	30°C
Detection:	UV at 210 nm
Retention times:	Amodiaquine = 1.2 minutes; artesunate = 4.3 minutes

Of the six samples of this ACT only the sample from Sanofi was a combination pill. The rest were packaged as distinct pills and no attempt was made to assay both APIs in a single HPLC run. The samples were weighed, manually crushed and, as described above, the claimed API content a weight% of API in the crushed material was used to calculate the amount of crushed material to be extracted for API analysis. Samples were sonicated for 20 minutes with 10 mL mobile phase and filtered prior to injection.

With this approach the content determined is based on the entire pill weight. An injection volume of 10 *u*L was used for artesunate solutions while 1 *u*L was used to quantitate amodiaquine. The formulations containing only artesunate showed a variety of unidentified components.

<u>Chromatogram of Sample for Artesunate Analysis</u>

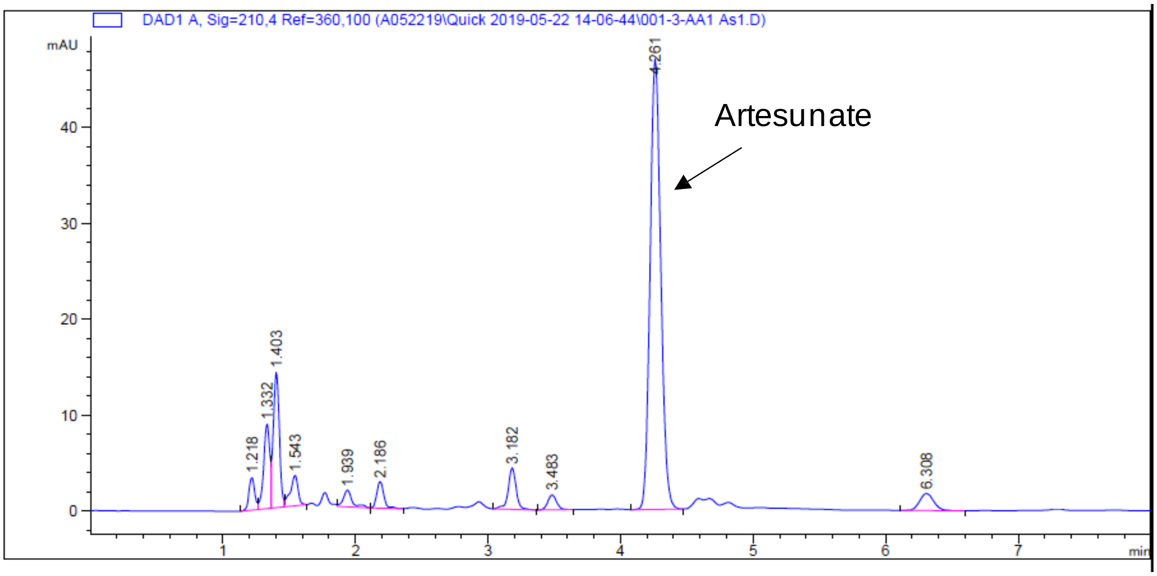

The pills containing only artesunate were much cleaner, as was the Sanofi combination pill.

<u>Chromatogram of Sanofi Sample for Amodiaquine Analysis</u>

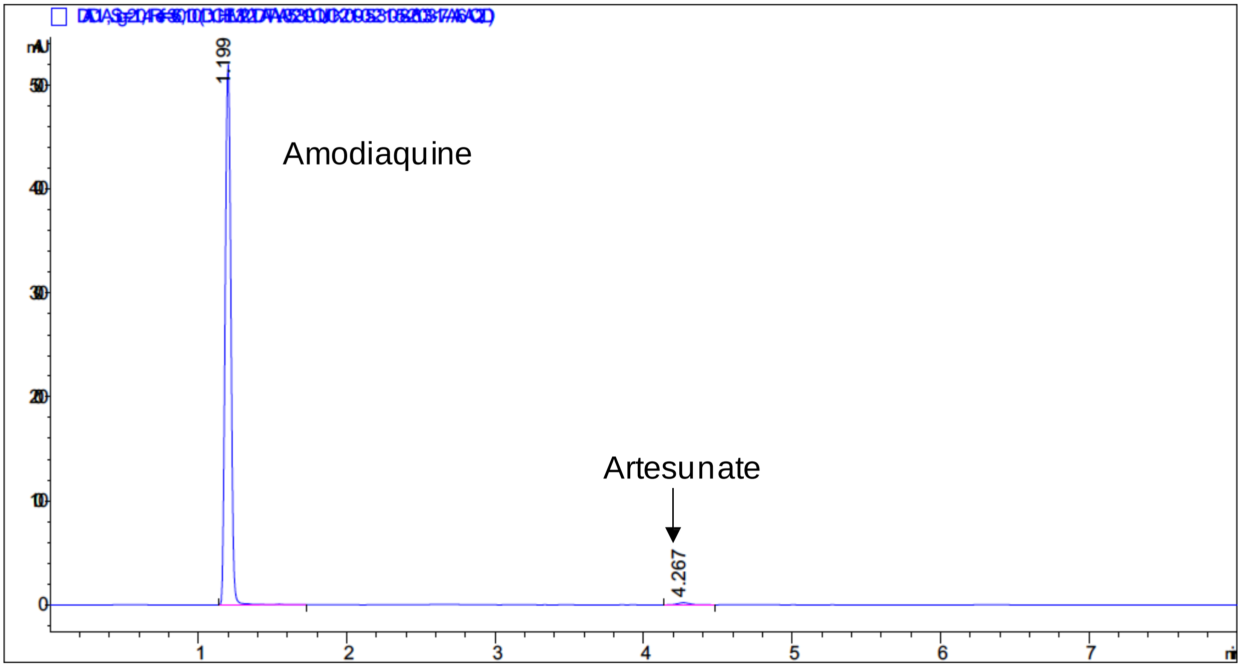

Table 6. Artesunate-Amodiaquine Results

Manufacturer	Brand	Lot #	Claim AQ	Claim AS	Found AQ	Found Ars	%AQ	% Ars
Front	Camosunate	110917	300	100	294.7	87.0	98.2%	87.0%
Front	Camosunate	120917	300	100	297.3	88.6	99.1%	88.6%
Front	Camosunate Pediatric	140917	75	25	74.7	20.7	99.6%	82.8%
Front	Camosunate Children	391117	150	50	152.7	44.5	101.8%	89.0%
Front	Camosunate Junior	1707818	300	100	292.9	95.4	97.6%	95.4%
Sanofi	ASAQ Winthrop	6MA455	270	100	278.9	95.8	103.3%	95.8%

Four of the six samples contained less than 90% of the label claim for artesunate. Amodiaquine samples were all close to claimed content.

The remaining eight samples contained piperaquine or piperaquine phosphate. Piperaquine is known to have solubility problems and show unpredictable behavior in HPLC analyses. With low pH mobile phases this compound does not elute even with high levels of organic modifier. At pH 4.8, 80% acetonitrile elutes this compound at 5.4 minutes. Using this a sample diluent it was found that piperaquine did not dissolve even at 1 mg/mL. It is necessary to use a higher pH (6.9) to achieve dissolution. Using 50% ACN vs. pH 6.9, piperaquine elutes at 5.1 minutes with a slight tail. Under these conditions artemisinin appears as a well-resolved peak at 3.2 minutes.

Artemisinin-Piperaquine Samples

HPLC Conditions

Column:	Agilent Extend-C18; 250 X 4.6 mm; 5 *u*M particles
Mobile phase:	(50:50) Acetonitrile:10 mM phosphate buffer at pH 7
Flow rate:	1.5 mL/min
Temperature:	30°C
Detection:	UV at 210 nm
Retention times:	Artemisinin = 3.2 minutes; piperaquine = 5.1 minutes

A single pill from each sample was weighed and then manually crushed. Based on the pill weight and the claimed API content a weight% of API in the crushed material was estimated and used to calculate the amount of crushed material to be extracted for API analysis. With this approach the content determined is based on the entire pill weight. Samples were sonicated for 20 minutes with 10 mL diluent and filtered prior to injection. Artemisinin analyses targeted 2.5 mg/mL in acetonitrile while piperaquine samples were prepared at 1 mg/mL of mobile phase.

Chromatogram of Artemisinin-Piperaquine Sample for Piperaquine Analysis

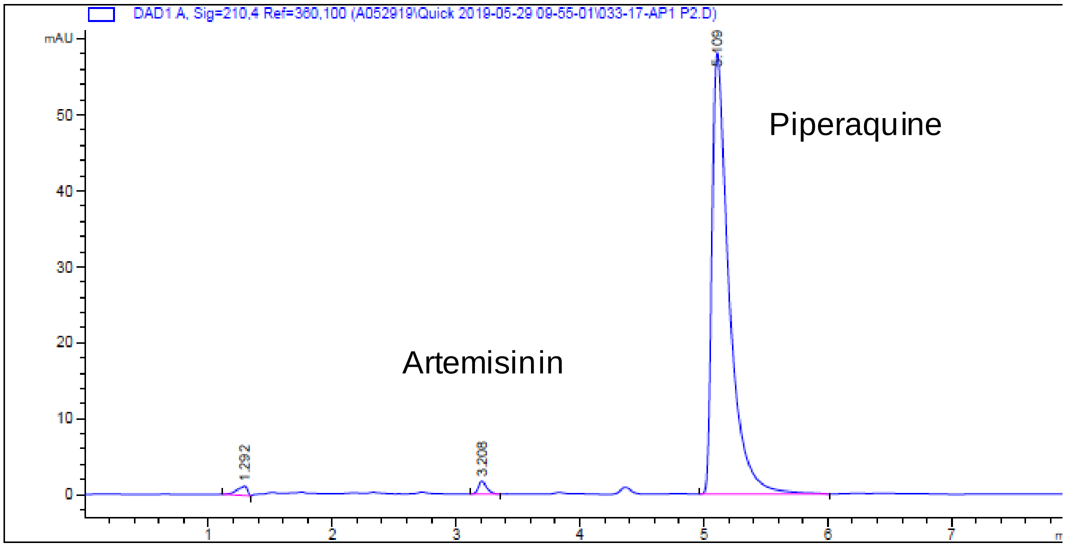

Chromatogram of Artemisinin-Piperaquine Sample for Artemisinin Analysis

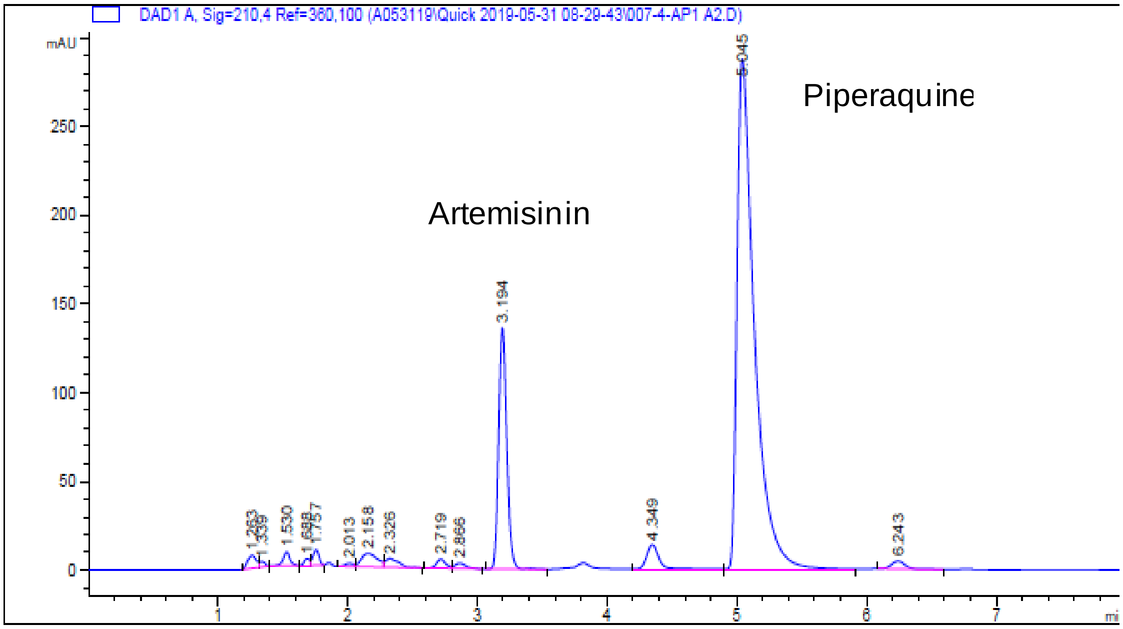

Using this method, the three artemisinin samples were quite close to the label claim. Piperaquine however was found to be extremely low. The label claimed piperaquine base rather than the phosphate typically specified. This would not be enough to account for the low amount of piperaquine found.

Table 7. Artemisinin-Piperaquine Results

Manufacturer	Brand	Lot #	Claim Art	Found Art	Claim Pip Q	Found PipQ	%Artemisinin	% Pip Q
Artepharm	Artequick	20180702	62.5	62.8	375	97	100.5	25.9
Artepharm	Artequick	20180702	62.5	61.9	375	119	99.0	31.7
Artepharm	Artequick	20170902	62.5	63.6	375	113	101.8	30.1

Dihydroartemisinin-Piperaquine Phosphate Samples

The analysis of dihydroartemisinin (DHA) is known to be problematic. DHA exists in two anomeric forms that interconvert during the HPLC analysis. This results in the observation of two peaks corresponding to the individual anomers, with a transition plateau between corresponding to interconverting molecules. Quantitation is then based on the summation of the area of the two peaks and the area between. The separation between anomers and the relative area of the transition region are affected by numerous chromatographic variables (pH, organic modifier, modifier level, temperature, sample age and flow rate). DHA is also known to have stability issues exacerbated by humidity and elevated temperature so it would not be surprising to find degradation of DHA in field ACT samples. Degradation is evidenced by the increased levels of its primary degradant. Degradation also occurs slowly in solution so observation of a small peak corresponding to the degradant is normal even with pristine DHA.

Initial conditions led to coelution of piperaquine with the first DHA anomer. Attempts to shift the retention away from the DHA anomers were unsuccessful leading to the compromise below, which limits the height of the interconversion plateau, while eluting the piperaquine in this region.

HPLC Conditions

Column:	Agilent Extend-C18; 250 X 4.6 mm; 5 *u*M particles
Mobile phase:	(60:40) Acetonitrile:10 mM phosphate buffer at pH 4.8
Flow rate:	1.5 mL/min
Temperature:	30°C
Detection:	UV at 210 nm
Retention times:	Dihydroartemisinin = 5.0 and 7.6 minutes; piperaquine = 6.7 minutes

A single pill from each sample was weighed and then manually crushed. Based on the pill weight and the claimed API content a weight% of API in the crushed material was estimated and used to calculate the amount of crushed material to be extracted for API analysis. With this approach the content determined is based on the entire pill weight. Samples were sonicated for 20 minutes with 10 mL diluent and filtered prior to injection. DHA analyses targeted 2.5 mg/mL in acetonitrile. Piperaquine phosphate levels were determined as described in the previous section.

Chromatogram of DHA Standard

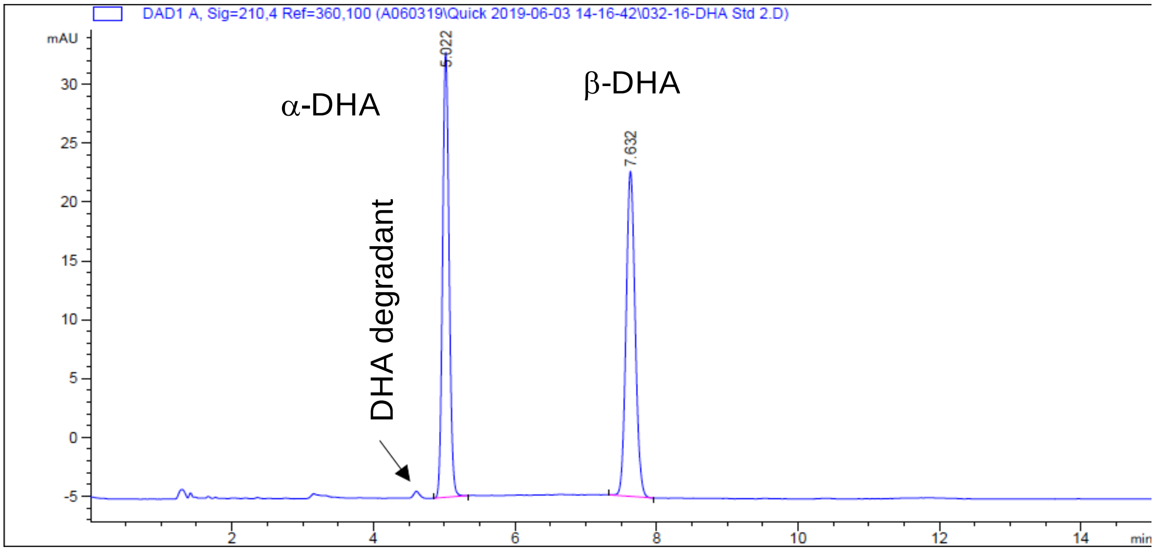

Chromatogram of DHA-Piperaquine Phosphate Sample

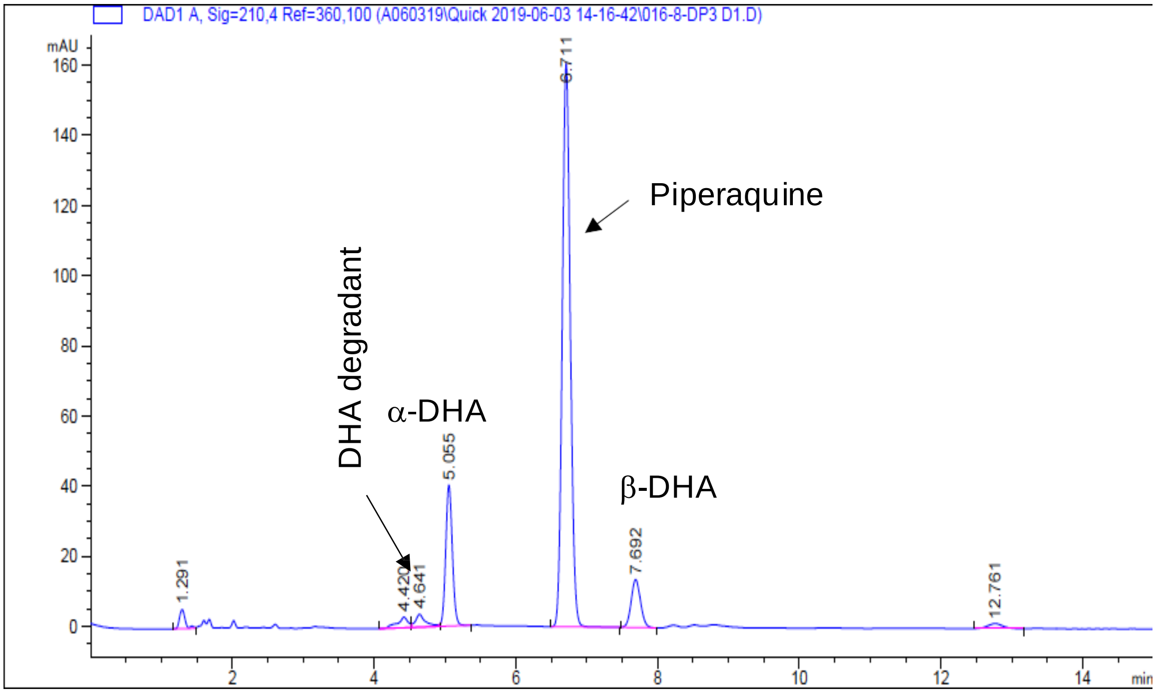

The DHA results were slightly low but not as bad as might be expected. DHA has been widely reported to have stability issues but only low levels of its degradant were observed in these samples. The degradant level was consistent with that observed in the reference material. All piperaquine phosphate results were in order.

DHA-Piperaquine Phosphate Sample Results

Manufacturer	Brand	Lot #	Claim DHA	Found DHA	Claim PipQ Phos	Found PipQ	% DHA	% PipQ
Bliss	P-Alaxin	H1AFN002	40	36.6	320	324	91.5	101.3
Bliss	P-Alaxin	H1AFN008	40	35.4	320	324	88.5	101.3
Bliss	P-Alaxin	H1AFN076	40	35.8	320	324	89.5	101.3
Bliss	P-Alaxin	H1AFN079	40	37.5	320	340	93.8	106.3
Bliss	P-Alaxin	H1AFN081	40	37.3	320	325	93.3	101.6
