# Supplementary material for: Assessing availability, prices, and market share of quality-assured malaria ACT and RDT in the private retail sector in Nigeria and Uganda
Source: Malar J. 2024 Feb 6;23:41. doi: 10.1186/s12936-024-04863-9 (PMC10848491; doi:10.1186/s12936-024-04863-9)
Supplement: Supplementary file 4 — Additional file 4. ACT Testing Results 2021. [file 12936_2024_4863_MOESM4_ESM.pdf]

Analysis of ACT Field Samples

April 15, 2022

Sarah L. Aleshire and Rodger W. Stringham

Workers for the Clinton Health Access Initiative (CHAI) collected various samples of Artemisinin Combination Therapy (ACT) packages from marketplaces in Nigeria. These samples were submitted to Medicines for All Institute (M4All) for analysis. CHAI was concerned that since the market had moved away from requiring SRA-qualified suppliers that many of the products might be fake or sub-standard. Samples were collected not in a representative way to draw conclusions regarding market integrity, but as a first pass to see if a problem might exist, justifying further investigation. As such the analysis of the samples did not need to be performed at a WHO PQ qualified laboratory. These samples were then submitted to M4All for preliminary evaluation. Reference samples of the APIs were kindly provided by Mangalam Drugs Company.

Table 1. Samples of DHA-Piperaquine phosphate ACT Formulations

Sample #	Manufacturer	Brand	Lot #	Claim DHA (mg)	Claim PipQ Phos (mg)
DP1	Bliss	P-Alaxin	J1AFN150	40	320
DP2	Bliss	P-Alaxin	J1AFN148	40	320

Table 2. Samples of Artemisinin-Piperaquine ACT Formulations

Sample #	Manufacturer	Brand	Lot #	Claim Arte (mg)	Claim PipQ (mg)
AP1	Artepharm	Artequick	20191104	62.5	375
AP2	Artepharm	Artequick	20200403	62.5	375

Table 3. Samples of Artesunate-Amodiaquine ACT Formulations

Sample #	Manufacturer	Brand	Lot #	Claim AQ (mg)	Claim AS (mg)
AA1	Geneith	Camosunate	450720	300	100
AA2	Geneith	Camosunate	541120	300	100
AA3	Geneith	Camosunate Junior	40220	300	100

Table 4. Samples of Artesunate-Mefloquine ACT Formulations

Sample #	Manufacturer	Brand	Lot #	Claim AQ (mg)	Claim AS (mg)
AM1	Acino	Artequin	20112156	250	200
AM2	Acino	Artequin	20112156	250	200

Table 5. Samples of Artemether-Lumefantrine ACT Formulations

Sample #	Manufacturer	Brand	Lot #	Claim ARM (mg)	Claim Lum (mg)
AL1	Shalina	Shal'Artem	1371593	20	120
AL2	Shalina	Shal'Artem	1371233	20	120
AL3	Meyer	Artelumex-Forte	692M2112X	80	480
AL4	Elbe	Amatem Forte	AL20007	80	480
AL5	Elbe	Amatem Forte	AL20016	80	480
AL6	Geneith	Coatal Tablets	210422	20	120
AL7	Novartis	Coartem	KY654	80	480
AL8	Novartis	Coartem	KAM96	80	480
AL9	Bliss	Lonart Tablets	GRT114	20	120
AL10	Bliss	Lonart Tablets	GRT135	20	120
AL11	Bliss	Lonart-DS	K1AFM035	80	480
AL12	Bliss	Lonart-DS	K1AFM054	80	480
AL13	Bliss	Gvither-Plus	A3AAF002	20	120
AL14	Embassy	Havax Forte	20HX44	80	480
AL15	Embassy	Havax	20HX89	20	120
AL16	Kesar	Fortune Saffron	T21315	20	120
AL17	Mzor	Lokmal QS-Combi	S063CBA	80	480
AL18	Bliss	Lonart Powder	K1AFM059	240	1440
AL19	Elbe	Amatem Forte Softgel	M21A027	80	480

The sample highlighted in yellow was identified as ‘dissolvable’ gel caps. The sample highlighted in green also claimed 500 mg of paracetamol.

Prior experience indicated that a single method would not address all formulation samples. The intent then became the development of four methods that could assay both components of each ACT simultaneously. This was expected to be complicated by the relative content of the APIs strongly favoring the base component, further exacerbated by the relatively low UV absorbance of the artemisinin-based APIs.

A review of the literature describing ACT analyses indicated that many authors ended up using C-18 columns with acetonitrile vs. pH 3 phosphate buffers. Previous work at CHAI laboratories indicated that the bases in the ACTs are strongly affected by mobile phase and sample diluent pH changes. It had also been observed that the HPLC peaks of the bases may overwhelm the peaks of the artemisinin derivatives such that resolution of these components may need to be better than otherwise expected. Preliminary tests of the various samples indicated that different assay conditions would be needed for each different ACT formulation.

Artemether-Lumefantrine Samples

HPLC Conditions

Column:	Agilent Extend-C18; 250 X 4.6 mm; 5 *u*M particles
Mobile phase:	(80:20) Acetonitrile:0.1% H_3PO_4 in water
Flow rate:	1.5 mL/min
Temperature:	30^oC
Detection:	UV at 210 nm
Retention times:	Lumefantrine = 2.0 minutes; artemether = 4.7 minutes

Separate sample preparations were made, with a target of 1 mg/mL for lumefantrine and 2 mg/mL for artemether. At the target concentration of the artemether, much of the lumefantrine base did not dissolve and the extract was filtered before analysis. This approach assumes that undissolved lumefantrine would not interfere with artemether dissolution and analysis.

A single pill from each sample was weighed and then manually crushed. Based on the pill weight and the claimed API content a weight% of API in the crushed material was projected and used to calculate the amount of crushed material to be extracted for API analysis. With this approach the content determined is based on the entire pill weight. Further, this approach assumes content uniformity rather than preparing a composite of several individual pills. Samples were sonicated for 20 minutes with 10 mL mobile phase and filtered prior to injection. Samples with a claim of 20 mg artemether were prepared by extracting an entire pill. An injection volume of 1 *u*L was used for lumefantrine assay while 10 *u*L was used for artemether.

The 'dissolvable' gel cap proved problematic as the gel portion did not completely dissolve in any solvent tested which allowed for API extraction. A sample was prepared by opening the pill and removing the contents. This provided detection of both APIs but required the assumption that the entirety of the claimed content was solubilized for solution comparison.

Paracetamol Sample

HPLC Conditions

Column:	Agilent Extend-C18; 250 X 4.6 mm; 5 *u*M particles
Mobile phase:	(30:70) Methanol:0.1% H_3PO_4 in water
Flow rate:	1.5 mL/min
Temperature:	30^oC
Detection:	UV at 210 nm
Retention time:	2.4 minutes

Sample was prepared at 1 mg/mL. A single pill was weighed and then manually crushed. Based on the pill weight and the claimed API content a weight% of API in the crushed material was projected and used to calculate the amount of crushed material to be extracted for API analysis. With this approach the content determined is based on

the entire pill weight. Samples were sonicated for 20 minutes with 10 mL methanol and filtered prior to injection. An injection volume of 1 *u*L was used.

Chromatogram of Sample for Lumefantrine Analysis

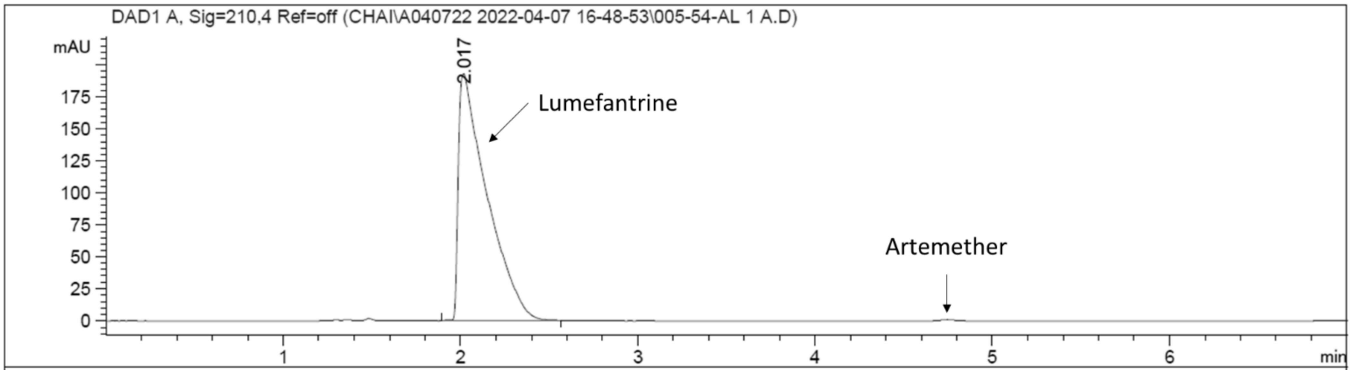

Chromatogram of Sample for Artemether Analysis

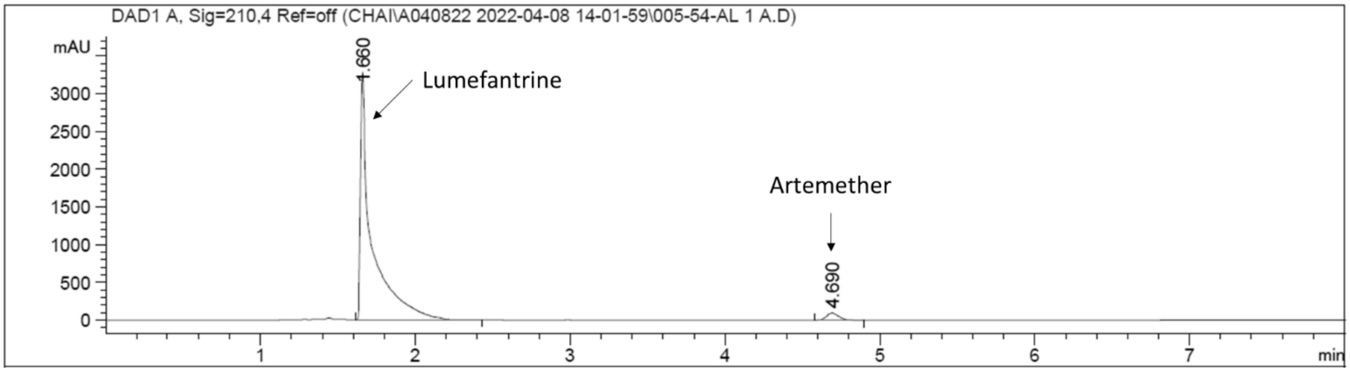

Chromatogram of Sample for Paracetamol Analysis

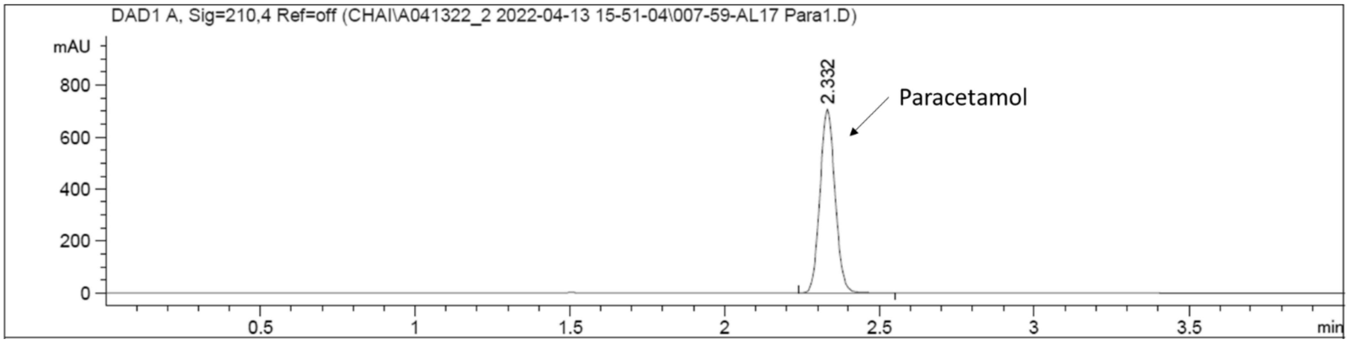

Almost all samples met their claimed content for both lumefantrine and artemether depending on the acceptability range. If 90% of label is considered passing two samples failed for both APIs including the softgel. The softgel result could be low due to inadequate solubility of the API in preparation or the possibility of API permeating into

the gel which was not included in the extraction. Due to these concerns, we cannot make a definite conclusion regarding the quality of this product

Table 6. Artemether-Lumefantrine Results

Manufacturer	Brand	Lot #	Claim Arm (mg)	Claim Lum (mg)	Found Arm (mg)	Found Lum (mg)	% Arm	% Lum
Shalina	Shal'Artem	1371593	20	120	20	125	101.3%	103.9%
Shalina	Shal'Artem	1371233	20	120	20	119	99.7%	98.8%
Meyer	Artelumex-Forte	692M2112X	80	480	82	489	102.2%	101.9%
Elbe	Amatem Forte	AL20007	80	480	79	481	99.0%	100.1%
Elbe	Amatem Forte	AL20016	80	480	79	467	98.8%	97.4%
Geneith	Coatal Tablets	210422	20	120	20	113	97.6%	94.0%
Novartis	Coartem	KY654	80	480	79	461	98.1%	96.0%
Novartis	Coartem	KAM96	80	480	81	479	101.0%	99.8%
Bliss	Lonart Tablets	GRT114	20	120	20	113	99.1%	94.4%
Bliss	Lonart Tablets	GRT135	20	120	20	121	98.8%	100.9%
Bliss	Lonart-DS	K1AFM035	80	480	78	461	97.4%	96.0%
Bliss	Lonart-DS	K1AFM054	80	480	77	440	96.2%	91.8%
Bliss	Gvither-Plus	A3AAF002	20	120	20	119	98.6%	99.3%
Embassy	Havax Forte	20HX44	80	480	79	453	98.8%	94.4%
Embassy	Havax	20HX89	20	120	20	124	101.4%	103.0%
Kesar	Fortune Saffron	T21315	20	120	16	95	80.9%	79.2%
Mzor*	Lokmal QS-Combi	S063CBA	80	480	81	500	101.2%	104.2%
Bliss	Lonart Powder	K1AFM059	240	1440	234	1300	97.4%	90.3%
Elbe	Amatem Forte Softgel	M21A027	80	480	1.1 mg/mL	0.6 mg/mL	56.7%	10.2%

*Found 469 mg of the claimed 500 mg which resulted in 93.8% of the claimed paracetamol content.

Artesunate-Amodiaquine Samples

HPLC Conditions
Column: Agilent Extend-C18; 250 X 4.6 mm; 5 *u*M particles
Mobile phase: (60:40) Acetonitrile:0.1% H_3PO_4 in water
Flow rate: 1.5 mL/min
Temperature: 30^oC
Detection: UV at 210 nm
Retention times: Amodiaquine = 1.2 minutes; artesunate = 3.8 minutes

All of the samples of this ACT were packaged as distinct pills and no attempt was made to assay both APIs in a single HPLC run. The samples were weighed, manually crushed and, as described above, the claimed API content a weight% of API in the crushed material was used to calculate the amount of crushed material to be extracted for API analysis. Samples were prepared at 1 mg/mL for both APIs. Samples were sonicated for 20 minutes with 10 mL mobile phase and filtered prior to injection. With this

approach the content determined is based on the entire pill weight. An injection volume of 10 *u*L was used for artesunate solutions while 1 *u*L was used to quantitate amodiaquine. Two samples containing artesunate and mefloquine were submitted as well. We do not have a reference standard for mefloquine so only the artesunate content was evaluated.

Chromatogram of Sample for Artesunate Analysis

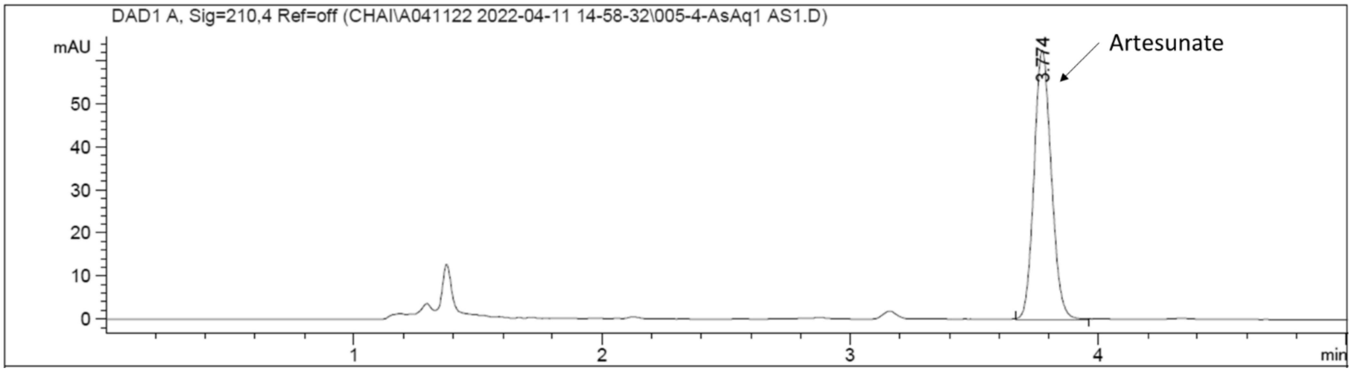

Chromatogram of Sample for Amodiaquine Analysis

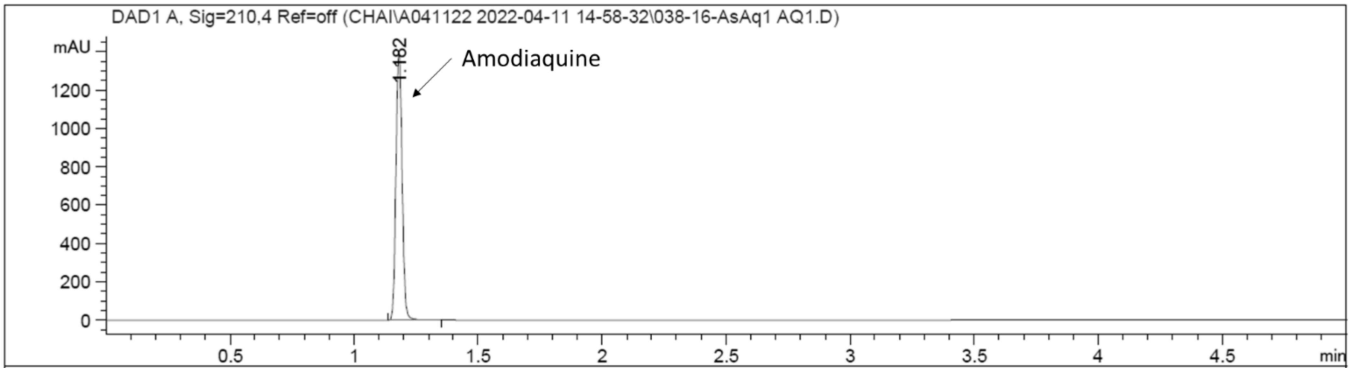

Table 7. Artesunate-Amodiaquine and Artesunate-Mefloquine Results

Manufacturer	Brand	Lot #	Claim AQ (mg)	Claim AS (mg)	Found AQ (mg)	Found AS (mg)	% AQ	%AS
Geneith	Camosunate	450720	300	100	322	94	107.3%	93.7%
Geneith	Camosunate	541120	300	100	318	98	106.0%	98.1%
Geneith	Camosunate Junior	40220	300	100	318	91	106.0%	91.3%

Manufacturer	Brand	Lot #	Claim MQ (mg)	Claim AS (mg)	Found MQ (mg)	Found AS (mg)	% MQ	%AS
Acino	Artequin	20112156	250	200	NA	198	NA	98.8%
Acino	Artequin	20112156	250	200	NA	197	NA	98.6%

Artemisinin-Piperaquine Samples

HPLC Conditions

Column:	Agilent Extend-C18; 250 X 4.6 mm; 5 *u*M particles
Mobile phase:	(70:30) Acetonitrile:10 mM phosphate buffer at pH 7
Flow rate:	1.5 mL/min
Temperature:	30°C
Detection:	UV at 210 nm
Retention times:	Artemisinin = 3.5 minutes; piperaquine = 5.8 minutes

A single pill from each sample was weighed and then manually crushed. Based on the pill weight and the claimed API content a weight% of API in the crushed material was estimated and used to calculate the amount of crushed material to be extracted for API analysis. With this approach the content determined is based on the entire pill weight. Samples were sonicated for 20 minutes with 10 mL diluent and filtered prior to injection. Artemisinin analyses targeted 2.5 mg/mL in acetonitrile while piperaquine samples were prepared at 1 mg/mL of mobile phase. An injection volume of 10 *u*L was used for artemisinin solutions while 5 *u*L was used to quantitate piperaquine.

Chromatogram of Artemisinin-Piperaquine Sample for Piperaquine Analysis

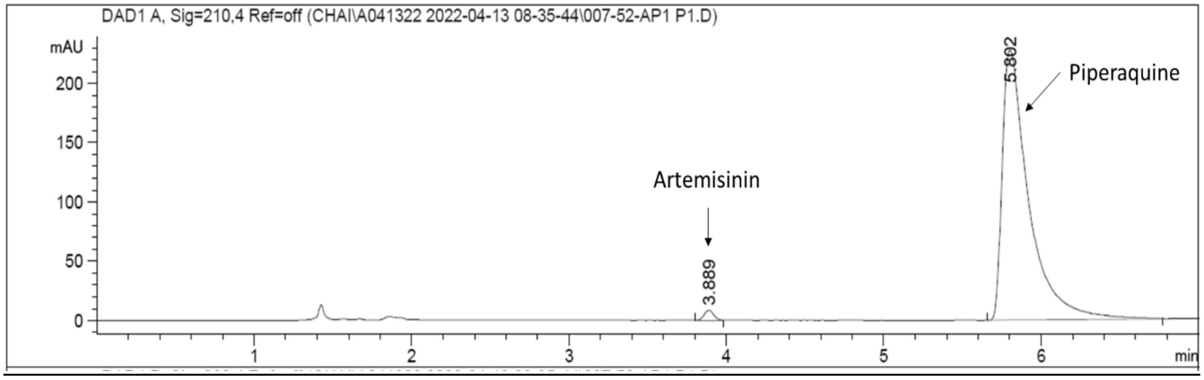

Chromatogram of Artemisinin-Piperaquine Sample for Artemisinin Analysis

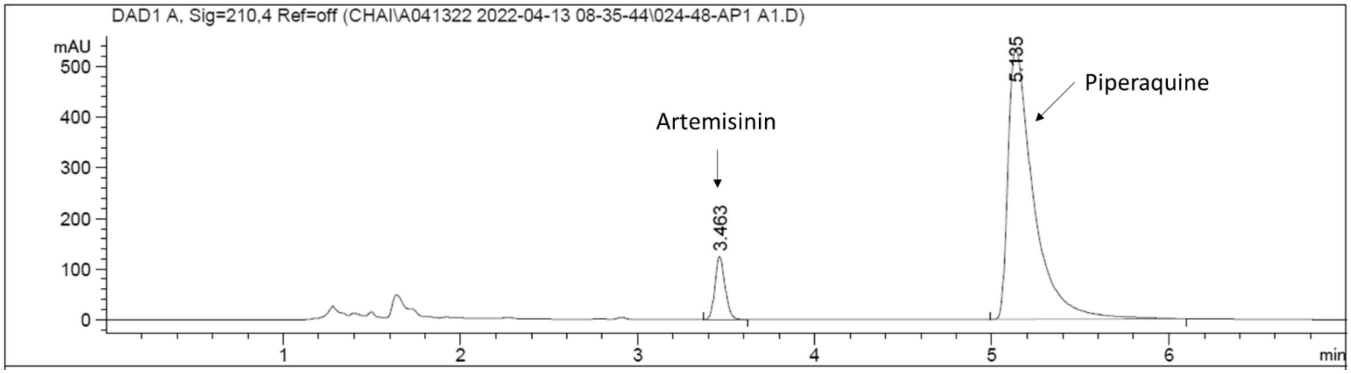

Artemisinin content was found to be consistent with the label claim while piperaquine was found to be extremely low in both samples. Similar results were obtained on three samples from the same manufacturer in 2019.

Table 8. Artemisinin-Piperaquine Results

Manufacturer	Brand	Lot #	Claim Arte (mg)	Claim PipQ (mg)	Found Arte (mg)	Found PipQ (mg)	% Artemisinin	% PipQ
Artepharm	Artequick	20191104	62.5	375	61.4	87	98.2%	23.3%
Artepharm	Artequick	20200403	62.5	375	62.6	91	100.2%	24.1%

Dihydroartemisinin-Piperaquine Phosphate Samples

The analysis of dihydroartemisinin (DHA) is known to be problematic. DHA exists in two anomeric forms that interconvert during the HPLC analysis. This results in the observation of two peaks corresponding to the individual anomers, with a transition plateau between corresponding to interconverting molecules. Quantitation is then based on the summation of the area of the two peaks and the area between. The separation between anomers and the relative area of the transition region are affected by numerous chromatographic variables (pH, organic modifier, modifier level, temperature, sample age and flow rate). DHA is also known to have stability issues exacerbated by humidity and elevated temperature so it would not be surprising to find degradation of DHA in field ACT samples. Degradation is evidenced by the increased levels of its primary degradant. Degradation also occurs slowly in solution so observation of a small peak corresponding to the degradant is normal even with pristine DHA.

HPLC Conditions

Column:	Agilent Extend-C18; 250 X 4.6 mm; 5 *u*M particles
Mobile phase:	(60:40) Acetonitrile:10 mM phosphate buffer at pH 4.8
Flow rate:	1.5 mL/min
Temperature:	30°C
Detection:	UV at 210 nm
Retention times:	Dihydroartemisinin = 3.2 and 4.3 minutes; piperaquine = 2.0 minutes

A single pill from each sample was weighed and then manually crushed. Based on the pill weight and the claimed API content a weight% of API in the crushed material was estimated and used to calculate the amount of crushed material to be extracted for API analysis. With this approach the content determined is based on the entire pill weight. Samples were sonicated for 20 minutes with 10 mL diluent and filtered prior to injection. DHA analyses targeted 2.5 mg/mL and piperaquine at 1 mg/mL. An injection volume of 10 *u*L was used for DHA solutions while 1 *u*L was used to quantitate piperaquine.

Chromatogram of DHA Standard

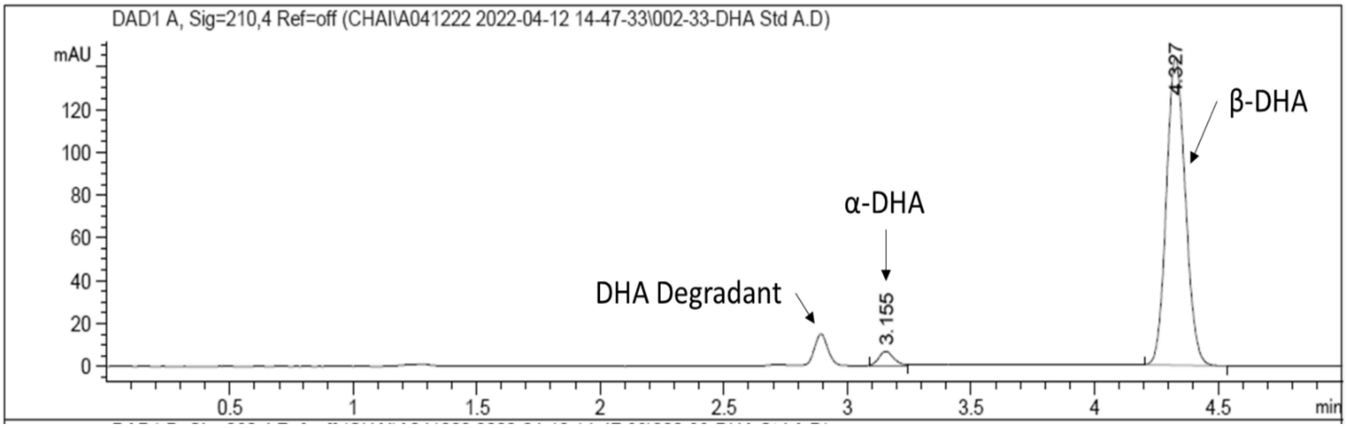

Chromatogram of DHA-Piperaquine Phosphate Sample for DHA Analysis

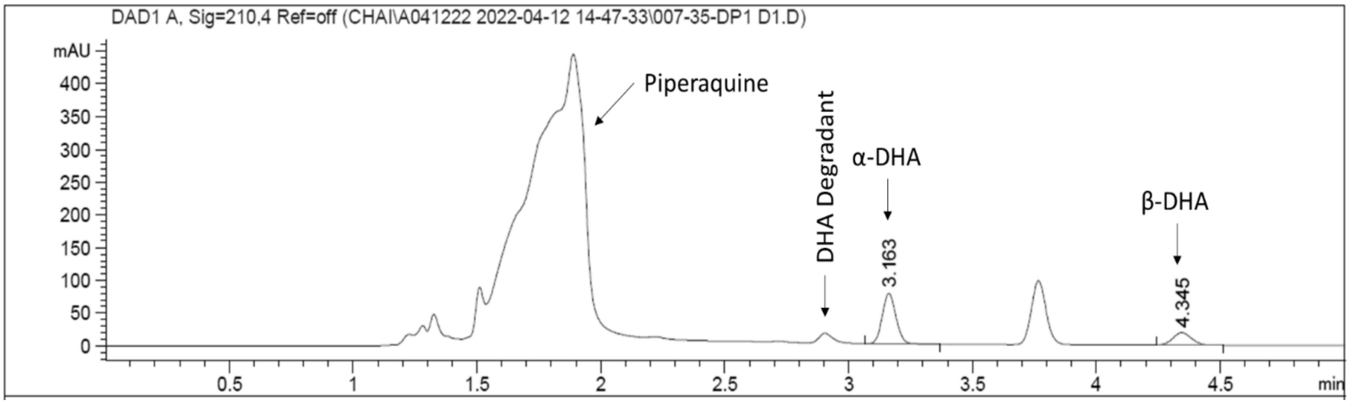

Chromatogram of DHA-Piperaquine Phosphate Sample for Piperaquine Analysis

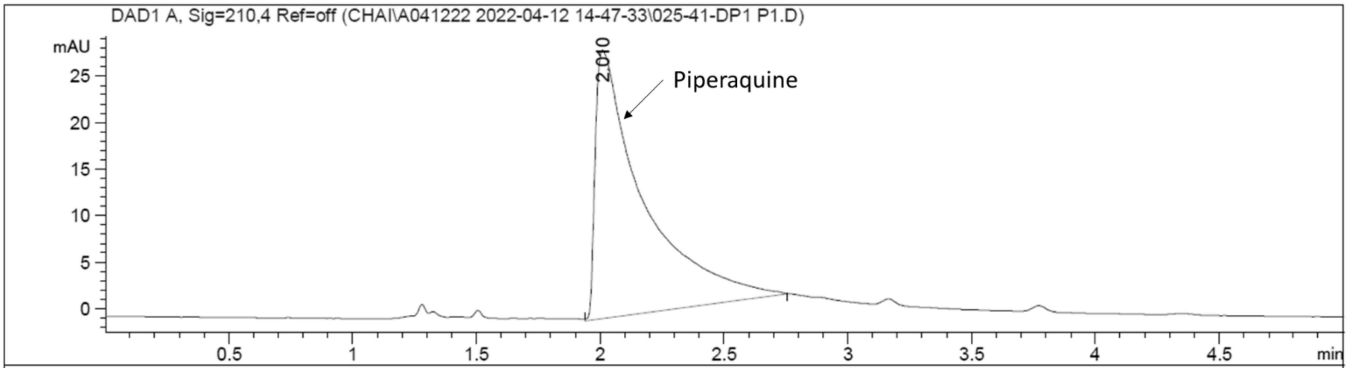

While the DHA content was as expected the piperaquine content was extremely low. Five samples of the same product were collected and analyzed in 2019. Those samples met specifications for both DHA and piperaquine.

Table 9. DHA-Piperaquine Phosphate Results

Manufacturer	Brand	Lot #	Claim DHA (mg)	Claim PipQ Phos (mg)	Found DHA (mg)	Found PipQ Phos (mg)	% DHA	% PipQ Phos
Bliss	P-Alaxin	J1AFN150	40	320	39	98	96.8%	30.6%
Bliss	P-Alaxin	J1AFN148	40	320	39	118	98.3%	37.0%
