# Supplementary material for: Assessing availability, prices, and market share of quality-assured malaria ACT and RDT in the private retail sector in Nigeria and Uganda
Source: Malar J. 2024 Feb 6;23:41. doi: 10.1186/s12936-024-04863-9 (PMC10848491; doi:10.1186/s12936-024-04863-9)
Supplement: Supplementary file 5 — Additional file 5. WHO-PQ-ACT availability by country and year. [file 12936_2024_4863_MOESM5_ESM.docx]

## Additional File 5: WHO-PQ-ACT availability in Nigeria and Uganda

|  | **Fraction with at least 1 WHO-PQ-ACT available on the day of the survey** | | | | | | | | |
| --- | --- | --- | --- | --- | --- | --- | --- | --- | --- |
|  | **2014/2016** | 2016 95% | 2016 95% | **2018*/2019** | 2018 95% | 2018 95% | **2020/2021** | 2021 95% | 2021 95% |
| **Nigeria** | **0.72** | 0.67 | 0.75 | **0.49** | 0.44 | 0.54 | **0.15** | 0.11 | 0.19 |
| Lagos | **0.83** | 0.78 | 0.87 | **0.71** | 0.64 | 0.76 | **0.12** | 0.08 | 0.17 |
| Kano | **0.57** | 0.5 | 0.64 | **0.33** | 0.27 | 0.4 | **0.16** | 0.12 | 0.23 |
| Urban | **0.8** | 0.75 | 0.84 | **0.54** | 0.49 | 0.6 | **0.14** | 0.1 | 0.19 |
| Rural | **0.55** | 0.47 | 0.62 | **0.32** | 0.22 | 0.43 | **0.16** | 0.1 | 0.25 |
| Drug Shop | **0.7** | 0.65 | 0.74 | **0.44** | 0.39 | 0.5 | **0.14** | 0.11 | 0.19 |
| Pharmacy | **0.84** | 0.71 | 0.91 | **0.74** | 0.62 | 0.83 | **0.2** | 0.1 | 0.36 |
| **Uganda** | **0.71** | 0.67 | 0.74 | **0.64** | 0.59 | 0.69 | **0.52** | . | . |
| Urban | **0.75** | 0.67 | 0.81 | **0.72** | 0.63 | 0.79 |  |  |  |
| Rural | **0.69** | 0.64 | 0.73 | **0.60** | 0.54 | 0.66 |  |  |  |
| Drug Shop | **0.66** | 0.60 | 0.70 | **0.65** | 0.58 | 0.71 | **0.50** | . | . |
| Pharmacy | **0.95** | 0.73 | 0.99 | **0.73** | 0.40 | 0.92 | **0.41** | . | . |
| Private clinic/doctor | **0.77** | 0.70 | 0.83 | **0.63** | 0.54 | 0.71 | **0.76** | . | . |
| Not-for-profit clinic | **0.83** | 0.58 | 0.95 |  |  |  |  |  |  |
