# Supplementary material for: Assessing availability, prices, and market share of quality-assured malaria ACT and RDT in the private retail sector in Nigeria and Uganda
Source: Malar J. 2024 Feb 6;23:41. doi: 10.1186/s12936-024-04863-9 (PMC10848491; doi:10.1186/s12936-024-04863-9)
Supplement: Supplementary file 6 — Additional file 6. Non-WHO-PQ-ACT availability by country and year. [file 12936_2024_4863_MOESM6_ESM.docx]

## Additional File 6: Non—WHO-PQ-ACT availability in Nigeria and Uganda

|  | **Fraction with at least 1 non-WHO-PQ-ACT available on the day of the survey** | | | | | | | | |
| --- | --- | --- | --- | --- | --- | --- | --- | --- | --- |
|  | **2014/2016** | 2016 95% | 2016 95% | **2018*/2019** | 2018 95% | 2018 95% | **2020/2021** | 2021 95% | 2021 95% |
| **Nigeria** | **0.13** | 0.10 | 0.16 | **0.56** | 0.51 | 0.61 | **0.54** | 0.48 | 0.59 |
| Lagos | **0.18** | 0.14 | 0.23 | **0.83** | 0.77 | 0.88 | **0.84** | 0.77 | 0.88 |
| Kano | **0.05** | 0.03 | 0.09 | **0.36** | 0.30 | 0.43 | **0.37** | 0.30 | 0.44 |
| Urban | **0.14** | 0.10 | 0.18 | **0.67** | 0.61 | 0.72 | **0.65** | 0.57 | 0.71 |
| Rural | **0.10** | 0.07 | 0.16 | **0.20** | 0.13 | 0.31 | **0.35** | 0.26 | 0.45 |
| Drug Shop | **0.09** | 0.07 | 0.12 | **0.50** | 0.44 | 0.55 | **0.52** | 0.46 | 0.57 |
| Pharmacy | **0.40** | 0.28 | 0.54 | **0.87** | 0.76 | 0.93 | **0.74** | 0.56 | 0.86 |
| **Uganda** | **0.06** | 0.04 | 0.08 | **0.40** | 0.35 | 0.45 | **0.55** | . | . |
| Urban | **0.11** | 0.07 | 0.17 | **0.42** | 0.33 | 0.50 |  |  |  |
| Rural | **0.04** | 0.02 | 0.06 | **0.39** | 0.33 | 0.46 |  |  |  |
| Drug Shop | **0.03** | 0.02 | 0.05 | **0.36** | 0.30 | 0.43 | **0.50** | . | . |
| Pharmacy | **0.50** | 0.30 | 0.70 | **0.55** | 0.26 | 0.81 | **0.44** | . | . |
| Private clinic/doctor | **0.08** | 0.04 | 0.13 | **0.46** | 0.38 | 0.55 | **0.81** | . | . |
| Not-for-profit clinic | **0.00** | 0.00 | 0.00 |  |  |  |  |  |  |
