# Supplementary material for: Assessing availability, prices, and market share of quality-assured malaria ACT and RDT in the private retail sector in Nigeria and Uganda
Source: Malar J. 2024 Feb 6;23:41. doi: 10.1186/s12936-024-04863-9 (PMC10848491; doi:10.1186/s12936-024-04863-9)
Supplement: Supplementary file 7 — Additional file 7. Mean price of WHO-PQ-ACTs by country and year. [file 12936_2024_4863_MOESM7_ESM.docx]

## Additional File 7: Average retail price of WHO-PQ-ACTs in Nigeria and Uganda

|  | **Volume-weighted mean price of WHO-PQ-ACT (Base Year USD, 2016 for Nigeria, 2014 for Uganda)** | | | | | | | | |  |
| --- | --- | --- | --- | --- | --- | --- | --- | --- | --- | --- |
|  | **2014/2016** | 2016 95% | 2016 95% | **2018*/2019** | 2018 95% | 2018 95% | **2020/2021** | 2021 95% | 2021 95% |  |
| **Nigeria** | **0.48** | 0.47 | 0.49 | **1.31** | 1.10 | 1.53 | **0.68** | 0.60 | 0.77 |  |
| Lagos | **0.73** | 0.71 | 0.74 | **1.59** | 1.44 | 1.74 | **2.37** | 1.46 | 3.27 |  |
| Kano | **0.32** | 0.31 | 0.33 | **0.72** | 0.61 | 0.83 | **0.63** | 0.58 | 0.67 |  |
| Urban | **0.58** | 0.57 | 0.59 | **1.44** | 1.26 | 1.62 | **0.77** | 0.53 | 1.00 |  |
| Rural | **0.34** | 0.33 | 0.36 | **0.70** | 0.56 | 0.84 | **0.62** | 0.55 | 0.69 |  |
| Drug Shop | **0.44** | 0.44 | 0.45 | **1.09** | 0.88 | 1.30 | **0.64** | 0.59 | 0.68 |  |
| Pharmacy | **0.76** | 0.72 | 0.80 | **1.67** | 1.45 | 1.90 | **3.06** | 2.00 | 4.11 |  |
| **Uganda** | **0.99** | 0.97 | 1.00 | **1.23** | 1.20 | 1.26 | **.** |  |  |  |
| Urban | **1.02** | 0.99 | 1.05 | **1.25** | 1.21 | 1.29 | **.** |  |  |  |
| Rural | **0.98** | 0.96 | 0.99 | **1.20** | 1.16 | 1.23 | **.** |  |  |  |
| Drug Shop | **0.82** | 0.80 | 0.83 | **1.07** | 1.05 | 1.10 | **.** |  |  |  |
| Pharmacy | **1.09** | 1.07 | 1.12 | **1.40** | 1.14 | 1.66 | **.** |  |  |  |
| Private clinic/doctor | **1.33** | 1.29 | 1.36 | **1.56** | 1.50 | 1.63 | **.** |  |  |  |
| Not-for-profit clinic | **0.55** | 0.50 | 0.60 |  |  |  | **.** |  |  |  |
