# Supplementary material for: Assessing availability, prices, and market share of quality-assured malaria ACT and RDT in the private retail sector in Nigeria and Uganda
Source: Malar J. 2024 Feb 6;23:41. doi: 10.1186/s12936-024-04863-9 (PMC10848491; doi:10.1186/s12936-024-04863-9)
Supplement: Supplementary file 8 — Additional file 8. Mean price of Non-WHO-PQ-ACTs by country and year. [file 12936_2024_4863_MOESM8_ESM.docx]

## Additional File 8: Average retail price of non-WHO-PQ-ACTs in Nigeria and Uganda

|  | **Volume-weighted mean price of non-WHO-PQ-ACT (Base Year USD, 2016 for Nigeria, 2014 for Uganda)** | | | | | | | | |  |
| --- | --- | --- | --- | --- | --- | --- | --- | --- | --- | --- |
|  | **2014/2016** | 2016 95% | 2016 95% | **2018*/2019** | 2018 95% | 2018 95% | **2020/2021** | 2021 95% | 2021 95% |  |
| **Nigeria** | **1.45** | 1.37 | 1.52 | **1.47** | 1.25 | 1.68 | **1.08** | 0.88 | 1.29 |  |
| Lagos | **1.73** | 1.66 | 1.80 | **1.73** | 1.48 | 1.97 | **1.59** | 1.47 | 1.71 |  |
| Kano | **1.05** | 0.92 | 1.19 | **1.03** | 0.87 | 1.18 | **0.72** | 0.66 | 0.79 |  |
| Urban | **1.71** | 1.63 | 1.78 | **1.50** | 1.27 | 1.73 | **1.27** | 1.02 | 1.51 |  |
| Rural | **1.13** | 1.00 | 1.26 | **0.90** | 0.80 | 0.99 | **0.67** | 0.56 | 0.78 |  |
| Drug Shop | **1.74** | 1.64 | 1.83 | **1.24** | 1.07 | 1.40 | **0.85** | 0.75 | 0.96 |  |
| Pharmacy | **1.30** | 1.20 | 1.39 | **1.96** | 1.65 | 2.26 | **1.76** | 1.62 | 1.89 |  |
| **Uganda** | **3.64** | 3.37 | 3.91 | **2.05** | 1.97 | 2.14 | **1.38** |  |  |  |
| Urban | **3.81** | 3.50 | 4.12 | **2.39** | 2.25 | 2.52 |  |  |  |  |
| Rural | **3.15** | 2.63 | 3.67 | **1.79** | 1.70 | 1.89 |  |  |  |  |
| Drug Shop | **2.78** | 2.17 | 3.38 | **1.75** | 1.64 | 1.86 | **1.53** |  |  |  |
| Pharmacy | **3.65** | 3.27 | 4.04 | **2.37** | 1.81 | 2.93 | **1.39** |  |  |  |
| Private clinic/doctor | **4.14** | 3.73 | 4.55 | **2.33** | 2.21 | 2.45 | **1.16** |  |  |  |
