# Supplementary material for: Assessing availability, prices, and market share of quality-assured malaria ACT and RDT in the private retail sector in Nigeria and Uganda
Source: Malar J. 2024 Feb 6;23:41. doi: 10.1186/s12936-024-04863-9 (PMC10848491; doi:10.1186/s12936-024-04863-9)
Supplement: Supplementary file 9 — Additional file 9: Table S6. Market share of WHO-PQ-ACTs by country and year. [file 12936_2024_4863_MOESM9_ESM.docx]

## Additional File 9: Market share of WHO-PQ-ACTs in Nigeria and Uganda

|  | **Fraction of doses of ACT sold that were WHO-PQ-ACT (weighted)** | | | | | | | | |  |
| --- | --- | --- | --- | --- | --- | --- | --- | --- | --- | --- |
|  | **2014/2016** | 2016 95% | 2016 95% | **2018*/2019** | 2018 95% | 2018 95% | **2020/2021** | 2021 95% | 2021 95% |  |
| **Nigeria** | **0.92** | 0.87 | 0.96 | **0.51** | 0.40 | 0.61 | **0.16** | 0.04 | 0.28 |  |
| Lagos | **0.88** | 0.82 | 0.95 | **0.52** | 0.43 | 0.62 | **0.06** | 0.01 | 0.11 |  |
| Kano | **0.94** | 0.88 | 1.00 | **0.48** | 0.25 | 0.70 | **0.23** | 0.29 | 0.43 |  |
| Urban | **0.92** | 0.87 | 0.97 | **0.47** | 0.37 | 0.57 | **0.12** | 0.01 | 0.24 |  |
| Rural | **0.91** | 0.83 | 0.99 | **0.77** | 0.49 | 1.04 | **0.25** | -0.05 | 0.55 |  |
| Drug Shop | **0.96** | 0.94 | 0.98 | **0.49** | 0.36 | 0.61 | **0.19** | 0.03 | 0.35 |  |
| Pharmacy | **0.67** | 0.47 | 0.86 | **0.55** | 0.40 | 0.69 | **0.09** | 0.01 | 0.16 |  |
| **Uganda** | **0.97** | 0.95 | 0.99 | **0.80** | 0.73 | 0.88 |  |  |  |  |
| Urban | **0.92** | 0.87 | 0.98 | **0.85** | 0.74 | 0.96 |  |  |  |  |
| Rural | **0.99** | 0.98 | 1.00 | **0.75** | 0.67 | 0.83 |  |  |  |  |
| Drug Shop | **0.99** | 0.98 | 1.00 | **0.85** | 0.76 | 0.94 |  |  |  |  |
| Pharmacy | **0.93** | 0.85 | 1.00 | **0.94** | 0.83 | 1.00 |  |  |  |  |
| Private clinic/doctor | **0.96** | 0.92 | 1.00 | **0.70** | 0.59 | 0.81 |  |  |  |  |
