# Supplementary material for: Assessing availability, prices, and market share of quality-assured malaria ACT and RDT in the private retail sector in Nigeria and Uganda
Source: Malar J. 2024 Feb 6;23:41. doi: 10.1186/s12936-024-04863-9 (PMC10848491; doi:10.1186/s12936-024-04863-9)
Supplement: Supplementary file 10 — Additional file 10: Table S7. RDT Availability by country and year. [file 12936_2024_4863_MOESM10_ESM.docx]

## Additional File 10: RDT Availability in Nigeria and Uganda

|  | **Proportion of PMRs with at least 1 RDT in stock** | | | | | | | | |  |
| --- | --- | --- | --- | --- | --- | --- | --- | --- | --- | --- |
|  | **2014/2016** | 2016 95% | 2016 95% | **2018*/2019** | 2018 95% | 2018 95% | **2020/2021** | 2021 95% | 2021 95% |  |
| **Nigeria** | **0.17** | 0.14 | 0.21 | **0.12** | 0.09 | 0.16 | **0.18** | 0.14 | 0.23 |  |
| Lagos | **0.09** | 0.06 | 0.13 | **0.03** | 0.01 | 0.06 | **0.02** | 0.00 | 0.05 |  |
| Kano | **0.28** | 0.23 | 0.35 | **0.18** | 0.14 | 0.24 | **0.27** | 0.21 | 0.34 |  |
| Urban | **0.12** | 0.09 | 0.16 | **0.07** | 0.05 | 0.11 | **0.12** | 0.08 | 0.17 |  |
| Rural | **0.28** | 0.22 | 0.36 | **0.27** | 0.18 | 0.37 | **0.28** | 0.20 | 0.38 |  |
| Drug Shop | **0.18** | 0.14 | 0.22 | **0.13** | 0.09 | 0.17 | **0.19** | 0.15 | 0.24 |  |
| Pharmacy | **0.16** | 0.09 | 0.29 | **0.08** | 0.04 | 0.19 | **0.03** | 0.01 | 0.20 |  |
| **Uganda** |  |  |  | **0.37** | 0.32 | 0.42 | **0.56** | . | . |  |
| Urban |  |  |  | **0.46** | 0.37 | 0.54 |  |  |  |  |
| Rural |  |  |  | **0.32** | 0.26 | 0.38 |  |  |  |  |
| Drug Shop |  |  |  | **0.21** | 0.16 | 0.27 | **0.34** | . | . |  |
| Pharmacy |  |  |  | **0.45** | 0.19 | 0.74 | **0.91** | . | . |  |
| Private clinic/doctor |  |  |  | **0.63** | 0.54 | 0.71 | **0.57** | . | . |  |
