# Supplementary material for: Assessing availability, prices, and market share of quality-assured malaria ACT and RDT in the private retail sector in Nigeria and Uganda
Source: Malar J. 2024 Feb 6;23:41. doi: 10.1186/s12936-024-04863-9 (PMC10848491; doi:10.1186/s12936-024-04863-9)
Supplement: Supplementary file 11 — Additional file 11: Table S8. Mean price of RDTs by country and year. [file 12936_2024_4863_MOESM11_ESM.docx]

## Additional File 11: Average retail price of RDTs in Nigeria and Uganda

|  | **Volume-weighted mean price of RDT (Base Year USD, 2016 for Nigeria, 2014 for Uganda)** | | | | | | | | |  |
| --- | --- | --- | --- | --- | --- | --- | --- | --- | --- | --- |
|  | **2014/2016** | 2016 95% | 2016 95% | **2018*/2019** | 2018 95% | 2018 95% | **2020/2021** | 2021 95% | 2021 95% |  |
| **Nigeria** | **0.66** | 0.66 | 0.66 | **0.21** | 0.13 | 0.30 | **0.31** | 0.28 | 0.34 |  |
| Lagos | **0.96** | 0.96 | 0.96 | **1.22** | . | . | **0.85** | 0.46 | 1.23 |  |
| Kano | **0.37** | 0.37 | 0.37 | **0.21** | 0.13 | 0.29 | **0.31** | 0.28 | 0.34 |  |
| Urban | **0.96** | 0.95 | 0.96 | **0.27** | 0.12 | 0.42 | **0.32** | 0.28 | 0.36 |  |
| Rural | **0.37** | 0.37 | 0.37 | **0.15** | 0.12 | 0.19 | **0.30** | 0.26 | 0.35 |  |
| Drug Shop | **0.71** | 0.71 | 0.72 | **0.22** | 0.13 | 0.30 | **0.31** | 0.28 | 0.34 |  |
| Pharmacy | **0.56** | 0.56 | 0.57 | **0.27** | . | . | **0.24** | . | . |  |
| **Uganda** |  |  |  | **0.75** | 0.72 | 0.77 | **0.79** |  |  |  |
| Urban |  |  |  | **0.90** | 0.87 | 0.94 |  |  |  |  |
| Rural |  |  |  | **0.59** | 0.57 | 0.61 |  |  |  |  |
| Drug Shop |  |  |  | **0.53** | 0.50 | 0.57 | **0.92** |  |  |  |
| Pharmacy |  |  |  | **1.39** | 1.33 | 1.45 | **0.73** |  |  |  |
| Private clinic/doctor |  |  |  | **0.76** | 0.73 | 0.79 | **0.84** |  |  |  |
